# A Half-Century of Supramolecular Thermodynamics: A Retrospective of Our Multidisciplinary Approach

**DOI:** 10.3390/molecules31183197

**Published:** 2026-09-10

**Authors:** Angela F. Danil de Namor, Nawal Al Hakawati

**Affiliations:** Laboratory of Thermochemistry, School of Chemistry & Chemical Engineering, University of Surrey, Guildford GU2 7XH, Surrey, UK; n.elhakawati@bau.edu.lb

**Keywords:** supramolecular chemistry, thermodynamics, applications, extraction, ion selective electrodes

## Abstract

This review synthesizes 50 years of thermodynamic research in supramolecular chemistry, focusing on the interactions of diverse receptors with ionic and neutral species across various media. By evaluating a selection of foundational publications, this work highlights the critical role of host–guest selectivity. Crucially, we emphasize that a deep understanding of fundamental thermodynamics, including binding constants, enthalpy, and entropy changes, is essential to rationally guide and optimize practical applications. In doing so, we used nuclear magnetic resonance (NMR), ultraviolet-visible (UV-Vis) spectroscopy, conductometry, potentiometry and titration microcalorimetry to characterize solution processes. We consider the scope and limitations of these techniques as well as the inherent limitations of the reaction media. We analyze how solution studies correlate with solid state profiles derived from X-ray diffraction (XRD) scanning electron microscopy (SEM), energy dispersive atomic X-ray spectroscopy (EDAX), thermogravimetric analysis and infrared (IR) spectroscopy. This foundational knowledge bridges theory and practice, driving applications in environmental remediation, such as targeted pollutant removal, and the development of highly sensitive, on-site sensing devices for real-time water monitoring. Finally, based on the historical trends and current gaps identified in the selected literature, we offer strategic suggestions for further research in this area.

## 1. Introduction

Supramolecular chemistry has developed far beyond its origins as a study of non-covalent interactions to become an integral part of modern material science and technology.

Unlike molecular chemistry (covalent bonds between atoms to give the molecule), supramolecular chemistry (a term introduced by Lehn in 1988 [1]) involves the interaction of two or more chemical species through noncovalent bonds. Expansion of this concept led to the formation of supramolecular self-assemblies, which have been the basis of nanostructures and new materials [2,3]. The strength of this field lies in its capacity for molecular recognition, transport, and transformation. In fact, the selective behavior of a macrocycle for one species relative to another can translate into recognition. It is through thermodynamics that a quantitative assessment of selectivity can be made. The conversion of a single ion into a complex one, in conjunction with the counter-ion, is a transformation process leading to the formation of new salts and, in solution, new electrolytes with physicochemical properties that differ significantly from those of common salts. To move from theory to application, thermodynamic data must be integrated to design strategies tailored for specific uses. The main aim of this retrospective review is to provide a comprehensive overview of our five-decade research program, tracing the roadmap of our investigations from core thermodynamic principles to diverse practical applications. The subsequent sections present the strategic approach of our investigations, categorizing the targeted supramolecular hosts alongside the core thermodynamic principles and insights derived from these systems.

## 2. Strategy of Research

**Scheme 1:**
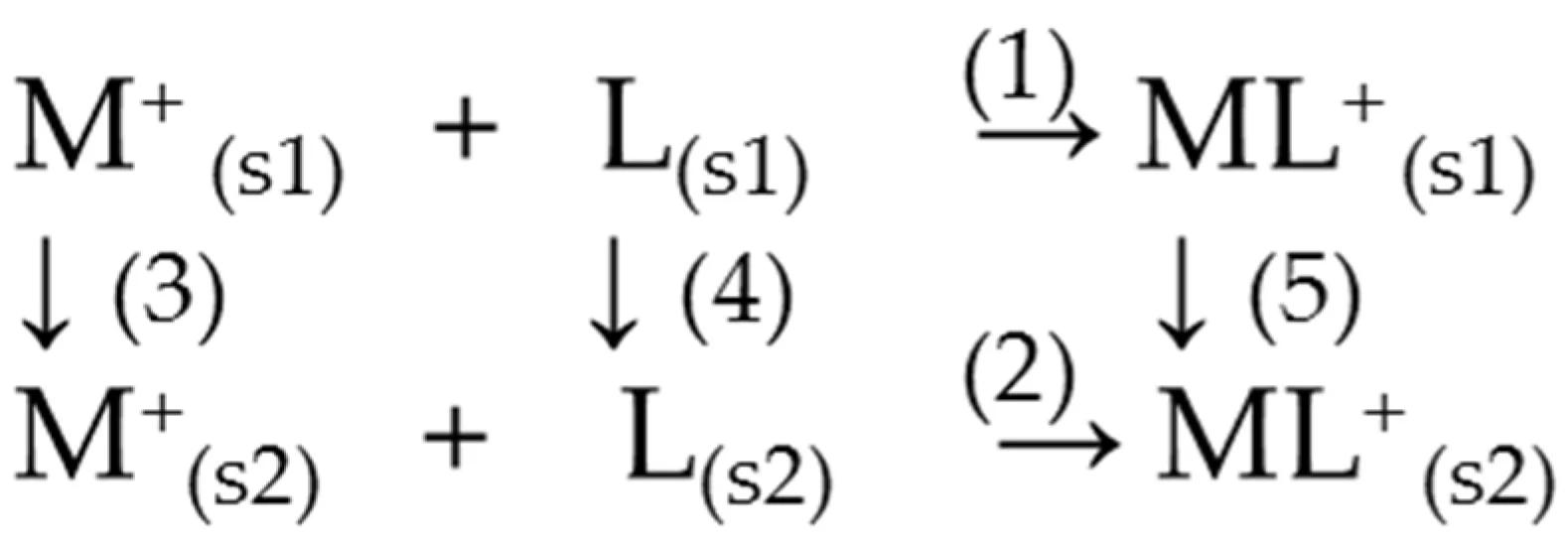
Thermodynamic cycle.

Historically, supramolecular thermodynamic studies have predominantly centered on the complexation process. These parameters include the following: (i) Gibbs energy of complexation (Δ*_c_G*^0^), which is related to the stability constant of the complex, *K_s_*, which reflects the strength of host–guest interactions; (ii) enthalpy (Δ*_c_H*^0^), which reflects the energetic nature of host–guest interactions, whether exothermic (favorable contribution to the Gibbs energy of the process) or endothermic; and (iii) entropy (Δ*_c_S*^0^), which provides insight into the predominant processes taking place, either complexation (conversion of two species into one, entropy loss, unfavorable contribution to the Gibbs energy of the process) or de-solvation or conformational changes (entropy gain, favorable contribution to the Gibbs energy of the process). In doing so, in our case, particular attention was placed on the limitations of the analytical techniques used for the determination of stability constants (hence, Δ*_c_G*^0^) and Δ*_c_H*^0^. Regarding the former, whenever possible, more than one analytical technique was used to derive these data. This parameter is crucial for determining the selectivity of a receptor for one species relative to another in each medium and temperature. Regarding enthalpy data, titration calorimetry was the approach adopted given the limitations of the use of the van’t Hoff isochore to obtain reliable enthalpy change values. However, a defining characteristic of our research is the comprehensive analysis of the solution thermodynamics of the metal cation, M^+^, the ligand, L, and the metal-ion complex, ML^+^, in reference solvent, s_1,_ and another solvent, s_2,_ as shown in Scheme 1.

In Scheme 1, Equations (1) and (2) are representative of the complexation process in each solvent, Δ*_c_P*° (*P* = *G*, *H*, *S*) in s_1_ and s_2_, while Equations (3)–(5) denote the transfer parameter, Δ*_t_P*° of the cation, ligand, and the metal-ion complex from the reference solvent to s_2_. Transfer Gibbs energies, enthalpies, and entropies were calculated from corresponding data of solutions of metal ion and metal-ion complex salts, and the ligand in each solvent to eliminate the contribution of the crystal lattice energies involved in solution processes. As such, transfer data provided information regarding the difference in solvation of reactants and product in the two solvents and assisted in assessing the role of solvation of the participating species in the complexation process [4,5,6,7,8]. For the calculation of single-ion values for the free and complex metal cations, the Parker convention [9] was used. For further information regarding the solution thermodynamics of 1:1 electrolytes and the calculation of single-ion values, readers are referred to previous publications by Danil de Namor and colleagues [10,11,12,13,14].

A further consideration in our studies was the calculation of the enthalpies of coordination, Δ*_coord_H*°, which refer to the process where the reactants and the product are in the pure physical state (solid, sol. or liquid, liq.) (Equation (1)) [15]. These data were obtained from the standard enthalpies of solution, Δ*_s_H*°, of the free MX and complex salts, MLX, the ligand, and the enthalpy of metal cation–ligand complexation, Δ*_c_H*°, in a given solvent (s) (Equation (2)):

MX (sol.) + L (liq. or sol.) → MLX (sol.)(1)

Δ*_coord_H*° = Δ*_s_H*° MX + Δ*_s_H*° L + Δ*_c_H*° − Δ*_s_H*° MLX (2)

We emphasized the importance of these data given that (i) they provide information about the enthalpic stability of the process in the absence of the solvent, and (ii) they are a reliable method to check the accuracy of the solution and complexation data used to derive the enthalpy of coordination. Indeed, for a given electrolyte and ligand, the Δ*_coord_H*° should be the same, independent of the medium used to obtain this data. (iii) It allows us to assess the counter-ion effect on the coordination process. The same thermodynamic cycle can be applied to the interaction of neutral macrocycles with neutral species. In this case, charges are omitted.

The research was conducted via a multi-technique approach. NMR spectroscopy was used as an initial screening tool to confirm host–guest complexation. If no interaction was detected, detailed thermodynamic studies were omitted. When complexation occurred, NMR was used to identify the active binding sites between the host and the guest. Conductometry was then used to evaluate the composition of the complex and investigate ion-pair formation. Then, calorimetry and potentiometry were used to determine the thermodynamic parameters described above (Scheme 1). Whenever possible, we isolated both, the free receptor and the resulting complexes for X-ray analysis, while thermogravimetry evaluated material stability. UV-visible spectrophotometry, SEM, and EDAX were utilized for sample analysis, morphology, and surface composition, respectively. Based on fundamental insights, we subsequently explored the application of these materials as decontaminating agents for water purification and for the development of sensing devices.

## 3. Selected Receptors

The receptors to be discussed in this review and their notations are shown in Table 1.

### 3.1. Cryptands

Lehn’s discovery of cryptands opened new thermodynamic research avenues [16,17,18,19,20]. We challenged the over-simplified view [3,4] that cavity–cation size matching alone drove selectivity, focusing instead on complexation and solution thermodynamics of reactants and the product in a variety of solvents.

Our goals were (i) to track solvation changes that reactants and product underwent upon complexation in moving from one solvent to another; and (ii) to clarify the controversy regarding the identity (Equation (3)) suggested by Villermaux and Delpuech [21] using cryptand 222, **1a** in the water–methanol (H_2_O-MeOH) solvent system leading to the formulation of a new extra thermodynamic convention for the calculation of single-ion values.

Δ*_t_G*° [Na^+^ 222] Cl^−^ = Δ*_t_G*° Cl^−^ (3)

Single-ion values cannot be experimentally determined because solutions must remain electrically neutral. To solve this, Parker’s convention [9], based on the tetraphenylarsonium tetraphenyl borate salt (Equation (4)) is currently used. This salt’s large ions shield their charges, allowing them to behave like neutral species:

Δ*_t_P*°(Ph_4_As^+^) _(H2O→s) =_ Δ*_t_P*°(Ph_4_B^−^) _(H2O→s)_ (4)

*P* = *G*. *H* .*S*

Schneider and colleagues [22] considered Equation (5) for a more direct calculation of single-ion values expressing the ‘cryptate convention’ in terms of Gibbs energies.

Δ*_t_G*° [M^+^ 222] (s_1_ → s_2) =_ Δ*_t_G*° [222] (s_1_ → s_2_) (5)

Despite limitations of the van’t Hoff equation [23], these authors used it for calculating heats of complexation for three cations in various solvents. Abraham and colleagues challenged this, stating that Equation (5) required the following identity (Equation (6)) to hold [24]:

Δ*_t_G*° [M_1_^+^ 222] (s_1_ → s_2) =_ Δ*_t_G*° [M_2_^+^ 222] (s_1_ → s_2_) (6)

Using data from Schneider, they carried out experimental work on the thermodynamics of **1a** and concluded that Equation (5) holds for the transfer of only a few metal-ion cryptates from water to *N*,*N*-dimethylformamide (DMF) or dimethyl sulfoxide (DMSO) but not to acetonitrile (MeCN) or propylene carbonate (PC). In addition, single-ion values from water differed significantly from those obtained by the Parker convention (Equation (4)). To address these discrepancies, we conducted a comprehensive calorimetric study by measuring the enthalpies of complexation of univalent cations and **1a**, as well as the enthalpies of solution of reactants and product across various dipolar aprotic solvents. This allowed us to evaluate solvent effects upon complexation and test the validity of the cryptate convention for calculating single-ion values. The outcome of this investigation sheds light on the driving force behind complex formation. Indeed, studies carried out in DMF, DMSO [24], MeCN, PC [8] and nitromethane (NM) [25,26,27]) showed in all cases that

Δ*_c_P*^0^ (s_2_) − Δ*_c_P*^0^ (s_1_) = Δ*_t_P*° M^+^ (s_1_ → s_2_) (7)

**Figure 1:**
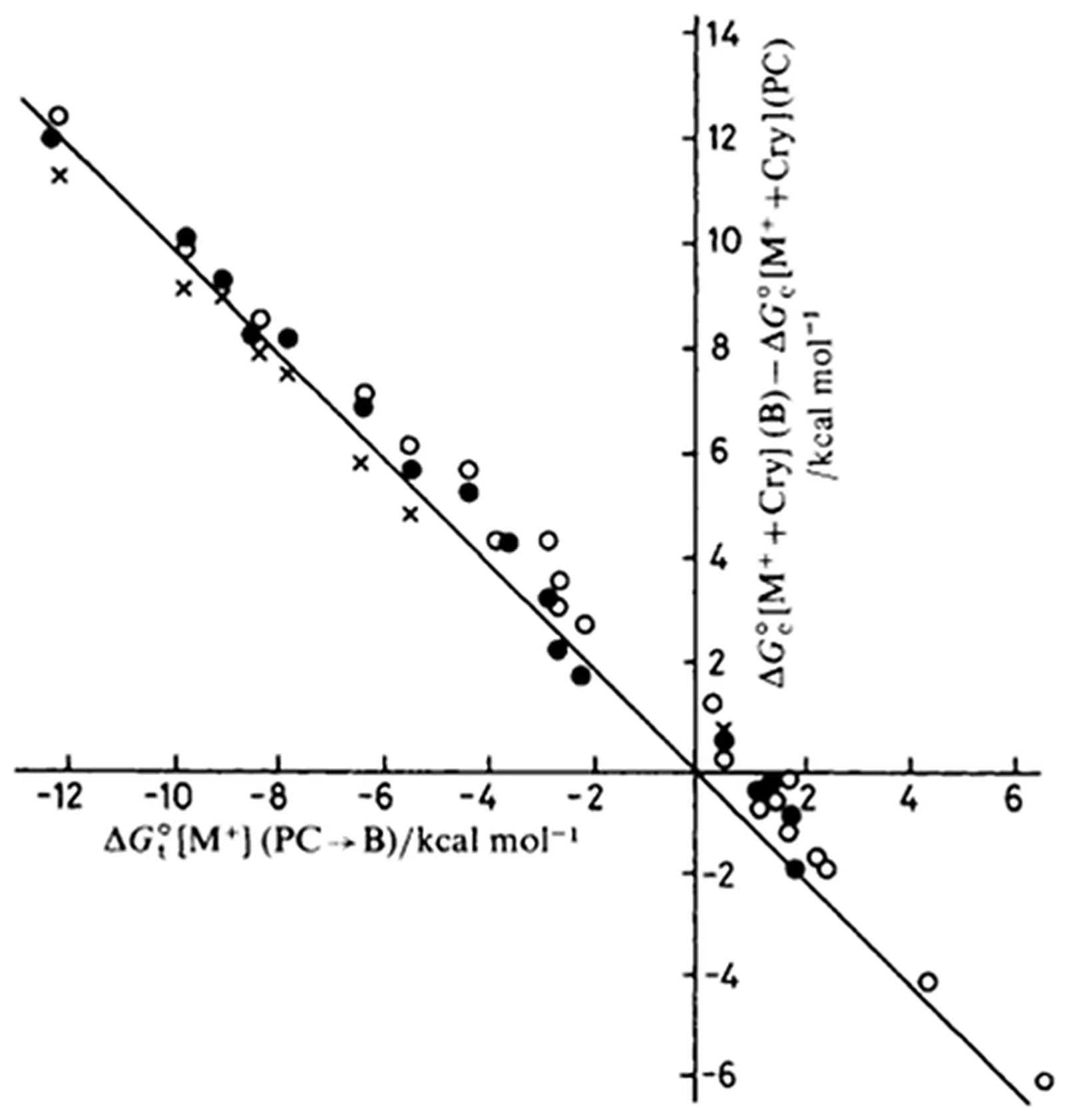
Plot of Δ*G*^0^*_c_*[M^+^ + Cry] (B) − Δ*G*^0^*_c_* [M^+^ + Cry] (PC) against single-ion Δ*G*^0^*_t_* [M^+^] (PC → B) based on the Ph_4_AsPh_4_B convention: x, cryptand 211, **2b**; ●, cryptand 221, **2a**; o, cryptand 222, **1a**.

In fact, the correlation between the difference in the thermodynamics of complexation of univalent cations and **1a** in two solvents and data for the transfer of the cation between these solvents when plotted in terms of Gibbs energies led to a straight line not only for **1a** but also for corresponding data reported in the literature for cryptands 221, **2a** and 211, **2b** [28,29], as shown in Figure 1. We found these results striking. They revealed the main role played by the solvation of the cation in complexation processes involving univalent cations and cryptands in dipolar aprotic media. Furthermore, it was shown by using Scheme 1 that the identities shown in Equations (6) and (7) also hold in terms of enthalpies for univalent cations, giving single-ion values in accord with those derived from the Parker convention (Equation (4)). The transfer parameters for the metal-ion cryptates are independent of the cation, and those for the transfer of the free receptor are within experimental error of zero.

Then, we proceeded with the calculation of the entropy of cryptate formation, Δ*_cf_S*° for the process described by Equation (8) [30], which involves the transfer of the cation from the gas phase into solution (solvation), followed by complexation Equation (9):

M^+^ (g) + L(s_1_) → ML^+^(s_1_) (8)

Δ*_cf_S*° = Δ*_solv_S*°[M^+^] + Δ*_c_S*° (9)

Given that Δ*_cf_S*° was found to be constant for all systems considered, Equation (10) holds in dipolar aprotic solvents:

Δ*_c_S*° = constant − Δ*_solv_S*° [M^+^] (10)

**Figure 2:**
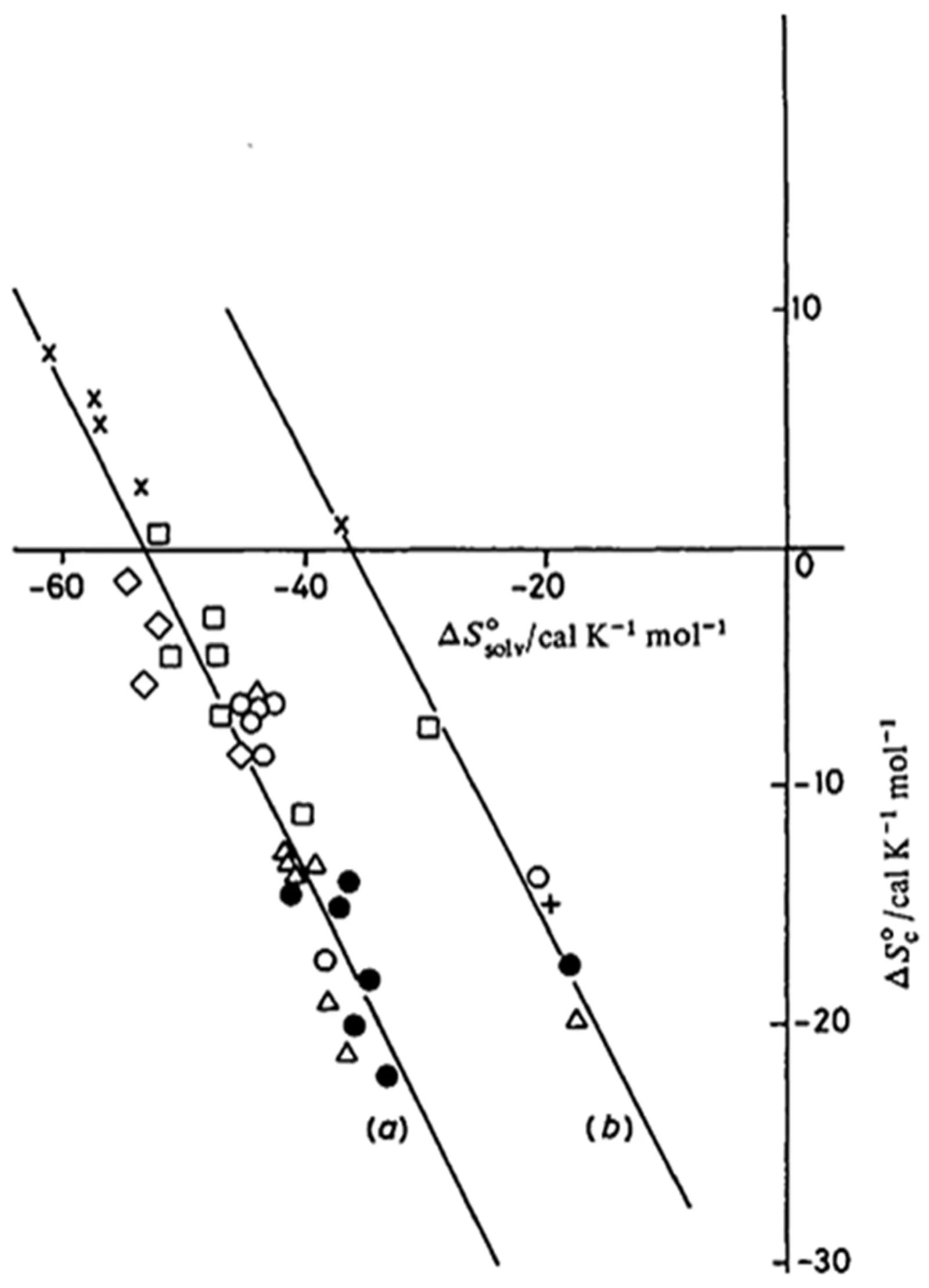
Linear correlation between entropies of complexation of cryptand 222, **1a** with metal-ion entropies of solvation of these ions (a) in non-aqueous solvents and (b) in water. x, Li^+^; □, Na^+^; o, K^+^; ∆, Rb^+^; ●, Cs^+^; ◊, Ag^+^; +, Ti^+^.

In fact, a plot of Δ*_c_S*° against ∆*_solv_S*° [M^+^] led to a straight line, as shown in Figure 2; its intercept gave a value for the entropy of cryptate formation, which was in excellent agreement with those obtained for individual cations in various solvents. Furthermore, the results clearly demonstrated that cations lost their solvation shells when entering the cavity of the receptor. Equation (10) was also tested in water (Figure 2). A straight line was obtained. As expected, the Δ*_cf_S*° was more favorable in water than in dipolar aprotic media, mainly due to the higher dehydration of the cation upon complexation with **1a**.

This research advanced our understanding of how solvation affects the complexation of bicyclic cryptands and univalent cations in dipolar aprotic solvents. It also highlights the importance of choosing receptors that corroborate findings with cryptand 222, **1a** while enabling future research into extracting univalent cations without environmentally hazardous nonaqueous solvents. For this purpose, dibenzo cryptand 222, **3** was selected because its phenyl groups allowed for easy polymerization via treatment with aldehyde in a basic medium. This was demonstrated using resins containing dibenzo-18-crown-6, **4** and their interactions with organic molecules [31] and for the extraction of 1:1 electrolytes from water [32]. Results obtained with DB222 [33,34] and univalent cations in different solvents followed the same pattern as those discussed above for 222.

The outcome of this research has settled the controversy regarding the use of the cryptate convention for the calculation of univalent single-ion values for their transfer among dipolar aprotic solvents. While studies on the role of solvation on the thermodynamics of complexation processes involving supramolecular hosts are very limited this issue must be explored further for other receptors.

Further research evaluated the use of the cryptate convention for the calculation of single-ion values for anions using metal(II) cryptate salts in dipolar aprotic media. Thus, two potassium cryptate salts containing two polarizable anions (tetraphenyl borate and picrate) alongside four alkaline earth metal (Mg(II), Ca(II), Sr(II) and Ba(II)) cryptates as perchlorates were isolated. Because deriving Gibbs energies of solution for bivalent ion salts is highly complex, the research was limited to the determination of enthalpy data by calorimetry in various solvents [35]. These results were striking: the transfer enthalpy data from water to DMF, DMSO, MeCN, and PC closely mirrored those derived from the Parker convention, with the implication emerging from these data that

Δ*_t_H*° [M^2+^ 222] (s_1_ → s_2_) = 0.0 ± 0.4 kJ/mol (11)

We concluded that further work is required to establish the universal validity of Equation (11) for bivalent metal-ion cryptates across a wide range of solvents. In fact, a detailed investigation on the interaction of Ba(II) and cryptand 222, **1a** in a variety of dipolar aprotic (DMF, DMSO, MeCN, PC) and protic solvents (water, MeOH) has shown that (i) the free barium electrolyte salt is more solvated than the complex one and (ii) the remarkable shielding effect provided by cryptand 222, **1a** for univalent cations weakens considerably for bivalent, and it is even more pronounced for tervalent cations [36].

Regarding lanthanide cations [37,38], we reported the thermodynamics of complexation of three lanthanide cations La(III), Pr(III) and Nd(III) with cryptands in MeCN and PC [39], followed by a study on the solution thermodynamics of several lanthanide cations (Eu(III), Gd(III), Tb(III), Ho(III), Er(III), Yb(III) and cryptand 222, **1a** in MeCN [40]. The first study provided valuable insights into favored entropy changes from cation desolvation, especially when handling ligands of varying cavity sizes, and enabled the calculation of single-ion transfer enthalpies between solvents. However, it was our second study, using a wide range of lanthanide cations that yielded deeper insights into the thermodynamics of lanthanide–cryptand 222 complexes.

**Figure 3:**
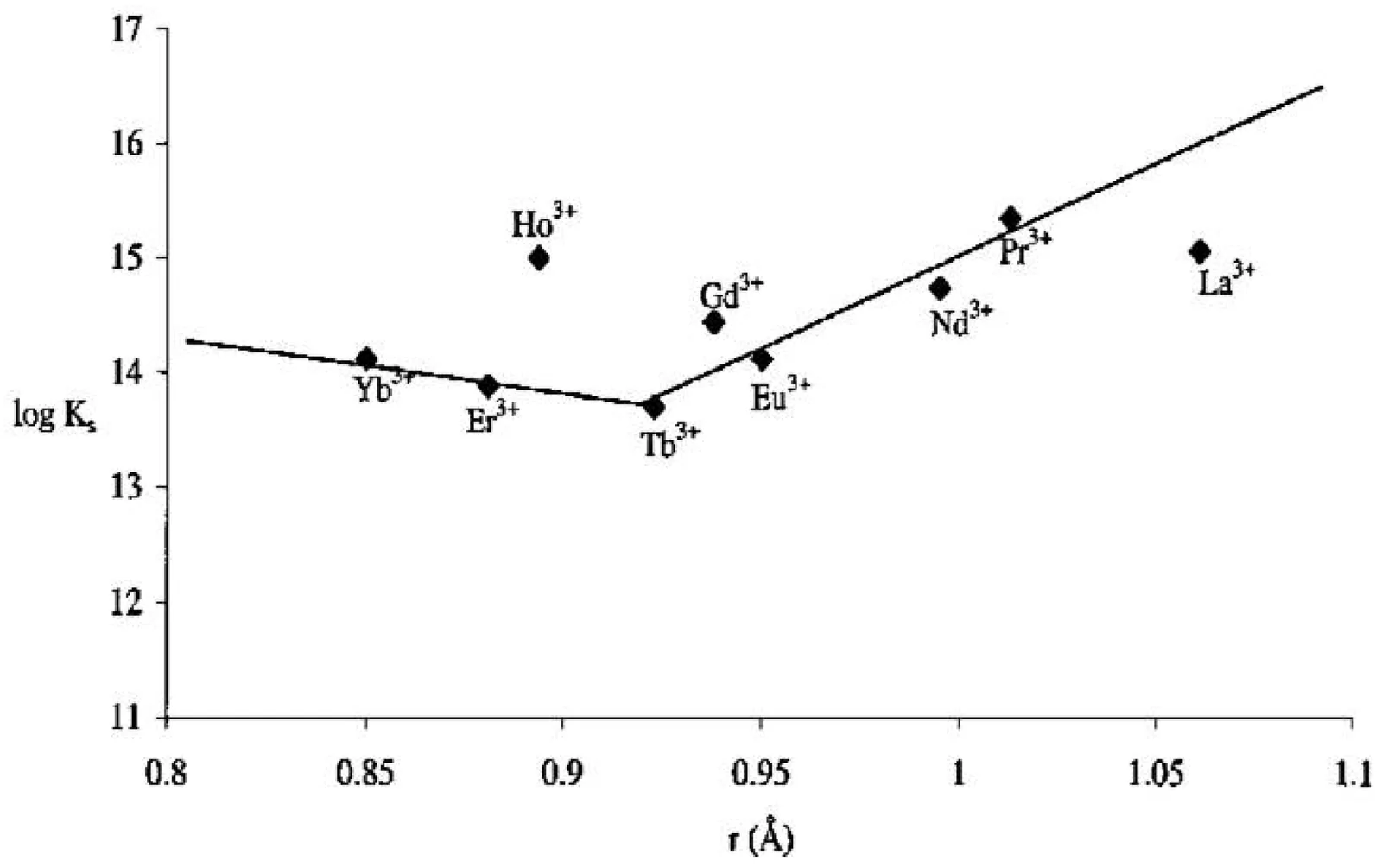
Stability constant (log*Ks*) of cryptand 222, **1a**–lanthanide complexes against cation radii in acetonitrile at 298.15 K.

The key finding of this study was that the stability constants (log *Ks*) of the lanthanide 222 complexes, determined *via* competitive titration microcalorimetry, can be successfully differentiated. This is vital as the ratio of these constants determines the receptor selectivity. Plotting log *Ks* vs. cation radii showed that stability decreases as cation size decreases from La(III) to Tb(III). However, a discontinuity occurs at Tb(III), after which the stability slightly increases, as shown in Figure 3. Stability constants (log *Ks*) of cryptand 222, **1a**-lanthanide complexes against cation radii in acetonitrile at 298.15 K. This pattern matches observations for other systems, as discussed in the original paper. We quantitatively assessed the remarkable medium effect on lanthanide–cryptand complexation when transitioning from MeCN to DMF. We strongly argued that some of the stability constants derived from potentiometry should be re-evaluated due to the high salt concentration and therefore the strong possibility of ion pair formation. In addition, the complexation process when dealing with lanthanide cations in dipolar aprotic media is kinetically slow. This was indeed the advantage of using a microcalorimeter designed to follow kinetically slow processes.

### 3.2. Crown Ethers

#### 3.2.1. New Coronand Salts and Their Scope for Use in Battery Technology

Solution studies of neutral macrocycles and ionic species yield complex ions that, alongside their counter-ions, form new salts. By considering the stability constants of these complexes, we isolated these salts and thermodynamically characterized their solution properties across various solvents. Our research demonstrated that converting a single ion into a complex one imparts properties that greatly differ from those of the bare ion, closely mimicking the behavior of hydrophobic ions [41]. Literature regarding the solution thermodynamics and applications of these electrolytes is notably scarce.

Rechargeable lithium batteries were and are the focus of intense research due to their vital role in electric vehicles. However, conventional electrolyte solutions present problems such as the low solubility of commonly used salts and the formation of ion pairs (non-conducting species) in solvents of medium or low permittivity. To overcome these limitations, we proposed using lithium coronand salts. We synthesized these salts using crown ethers known to be selective for lithium, such as 1-aza-12-crown-4, **5** and 15-crown-5, **6** [41,42] paired with highly polarizable anions. This investigation yielded three main conclusions:

(I) Thermodynamic analysis confirmed that the resulting complexes were stable enough for isolation (log *Ks* values for **5** and **6** and lithium salts range from 4.44 to 4.23 in acetonitrile) [15,41].
(II) Calorimetric data revealed that complex lithium salts underwent less solvation than standard lithium salts. This lower solvation corresponded to an increase in ionic conductivity (from 20.00 S.cm^2^.mol^−1^ for Li triflate to 23.5 S.cm^2^.mol^−1^ for the complex Li**6** triflate in PC and similar increases for other lithium salts), a critical property for lithium battery electrolytes.
(III) Coordination enthalpies [15] not only verified the accuracy of the solution and complexation data derived from different solvents but also reflected the anion effect on the coordination process.

Although supramolecular salts have seen notable applications in recent years [41,42,43,44,45,46,47,48,49,50,51,52], little progress has been made on the transition from thermodynamics of these salts to their applications. The use of new salts derived from Supramolecular Chemistry needs to be further explored in lower permittivity media where uncomplexed salts are characterized not only by their lower solubility but also by the formation of non-conducting species (ion pairs).

#### 3.2.2. Crown Ethers and Cryptands and Their Interactions with Amino Acids: The Enthalpy–Entropy Compensation Effect

While macrocyclic chemistry involving crown ethers and cryptands has mainly focused on cation complexation, the ability of crown ethers and cryptands to form hydrogen bonds with ammonium salts [53,54] offered an alternative pathway for molecular recognition. Building on the observation that 18-crown-6, **7** and cryptand 222, **1a** enhanced amino acid solubility in alcohols (MeOH, EtOH, 2-PrOH), we isolated and thermodynamically characterized seven amino acid-coronand and seven amino acid- cryptand 222 complexes [55,56]. Transfer Gibbs energies showed that the amino acid guest and the isolated complexes were fully exposed to the solvent. Complexation took place through hydrogen-bond formation between the protonated amino component of the zwitterionic amino acid and the donor atoms of the ligand. This statement was supported by experiments carried out in which the proton of the amino acid was blocked by an acetyl moiety, where no complexation was found in MeOH or EtOH. Computer calculations showed that the three hydrogen atoms of the amino acid were close enough to the crown ether as to enter hydrogen bonding with the three oxygen atoms of the ligand. The suggestion provided was in accord with X-ray diffraction studies previously reported for complexes of ammonium salts and crown ethers [57]. Interestingly, complex stability remained rather constant within the experimental error across these ligands Thus the stability constants of eighteen amino acids with 18-crown-6, **7** in MeOH were log*K_s_* = 3.2 ± 0.3 and in EtOH, log*K_s_* = 3.5 ± 0.2 and those for seventeen amino acids with cryptand 222, **1a** in MeOH were 3.5 ± 0.4 and in EtOH 3.3 ± 0.1 due to a striking enthalpy–entropy compensation effect. This was evidenced by linear plots of enthalpy vs. entropy for these amino acid complexes in these alcohols. Grunwald and Steel [58] noted that this enthalpy *vs.* entropy plot was a textbook example of compensation and solvent reorganization.

### 3.3. Cyclodextrins

Due to their lack of toxicity, cyclodextrins (CDs), which are essentially polymers of glucose, are widely used across food and pharmaceutical sectors with their history and applications extensively reviewed in the literature [59,60,61,62,63]. Our research focused on the following:

(i) Evaluating CDS, **8** and **10** as antibody models [64] by examining [64,65,66,67] their interactions with various azo-benzoate and substituted azo-benzoate haptens. To our knowledge, this work represented the first comprehensive thermodynamic investigation into the host properties of CDs toward haptens. By integrating the solution properties of these haptens across diverse solvents [64,65,66,67], we successfully interpreted the underlying binding process, notably, the solvent effect. In water, the guest molecule penetrates the receptor hydrophobic cavity to give inclusion or axial complexes, whereas in DMF, the solvent occupied the cavity restricting the guest to external interactions with the surface hydroxyl groups to form equatorial complexes [64].
(ii) The effect of β-CD, **9** on the transfer of drugs (sulphonamides) from water to a membrane-like solvent (Chloroform) [68].

Drug bioavailability depends mainly on the solubility of the drug in water and its transfer across the membrane. While CDs are used to enhance the solubility of poorly soluble drugs [69,70,71], this study evaluated the transfer thermodynamics of sulphonamide-CD complexes across a water/chloroform interface using chloroform as a membrane-like solvent. By combining transfer Gibbs energy data for free sulphonamides, CDs, and the drug–CD complex, we established that host–guest complexation does not take place within the chloroform phase. Isolation of the complex confirmed that the organic-phase drug solubility was that of the free drug. Consequently, the overall phase transfer process involved two steps: (i) the dissociation of the complex in water; and (ii) the transfer of the drug from water to chloroform. Given that, in the presence of CD, the extraction constant, *Kex* = (1/*Ks*) *Kt*, it follows that *Kt* ˃ *Kex*. Thermodynamically, this demonstrates that sulphonamides transfer into membrane-like solvents is more favorable in the absence of cyclodextrins than in their presence. Further kinetic studies are required to fully elucidate the actual transfer rates and mechanisms of these systems.

(iii) Cyclodextrin–Drug (Aspirin and Related Compounds) Interactions [72]

Building on our interest in the removal of pharmaceuticals from water, this study presented fundamental solution thermodynamics of commonly used pharmaceutical compounds (acetyl salicylic acid, 4-acetoxybenzoic acid, 5-acetyl salicylic acid) in water and *N*,*N*-dimethylformamide (DMF) at 298.15 *K* through solubility, conductance and calorimetric measurements. Ion-pair formation in water was determined to calculate the Gibbs energy of solution. Because these compounds are strongly solvated in DMF, only enthalpy data were reported. Special emphasis was placed on two distinct processes that occurred in aqueous solution: at lower pH, the drug is non-dissociated and characterized by an association constant, whereas at higher pH, ions predominate and an overall stability constant (*Ks*) was obtained. In DMF, ion pair formation was observed for both, the free and complexed drugs.

(iv) Cyclodextrin–monosaccharide interactions [73]

The aim of this research was to obtain a quantitative evaluation of the selectivity of α, **8** and β-CDs, **9** for monosaccharides in water using titration microcalorimetry and ^13^C NMR studies. Blocked monosaccharides were used to establish active sites of host–guest interactions. It was shown that the interaction of the receptor with the monosaccharide decreases significantly when moving from α- to β-CD. The weak complex stability is governed by extensive monosaccharide dehydration, yielding a favorable entropy contribution. Because of near-complete enthalpy–entropy compensation, CDs cannot achieve selective recognition of monosaccharides in water.

### 3.4. Calix[n]arene (n = 4, 6, 8)-Based Receptors

The interest surrounding calixarenes, particularly calix[4]arenes, stems from key synthetic work regarding phenol–formaldehyde reactions [74,75,76]. However, the one-step synthesis pioneered by Gutsche and colleagues [77,78,79,80,81] catalyzed intensive research, leading to a proliferation of scientific literature [82,83,84,85]. The versatility of these receptors has since driven multidisciplinary applications [86,87]. Unquestionably, calixarene chemistry has expanded the broader field of macrocyclic chemistry while providing opportunities to explore the thermodynamics of these systems as detailed below.

#### 3.4.1. Parent Calixarenes–Amine Interactions: From Neutral Species to New Electrolytes

While multiple research groups have explored the interactions between parent calixarenes, **11a**, **11b**, **11c** and amines [88,89,90,91,92], our laboratory has contributed several distinct insights to this field [93,94,95,96]. Although we have previously discussed these contributions in a critical review [97], the focus here is on the specific features that set our approach apart from prior studies. Notably, we used a benzonitrile–water immiscible solvent for the selective extraction of amines from water using parent calix[n]arenes (*n* = 4, 6, 8). Our work also expanded the scope of this chemistry to include macrocyclic amines such as cryptands 22, **1b** and 222, **1a** [93]. Furthermore, we investigated the solution thermodynamics and dissociation constants of calix[n]arenes in benzonitrile, used conductivity as the most effective tool to distinguish between hydrogen bonding and proton transfer, and reported the individual processes driving the overall extraction of amines by calixarenes.

#### 3.4.2. Calixarene Esters, Ketones and Amides and Their Interaction with Metal Cations

In 1991, we reported our initial finding on the calix[4]arene ester derivative, *p*-*tert*-butylcalix[4]arene tetraehanoate, **12**. This receptor possesses a hydrophobic cavity between its phenyl rings and a hydrophilic cavity formed by the functionalized phenolic oxygens. By studying its interactions with alkali–metal cations in MeOH and MeCN, we demonstrated that the inclusion of MeCN within the hydrophobic cavity produced a synergistic effect. This phenomenon pre-organizes the hydrophilic cavity for enhanced cation binding, resulting in higher complexation in MeCN compared to MeOH [98]. We further explored this allosteric effect in subsequent studies in MeCN and PhCN [99,100]. Strikingly, extraction experiments targeting alkali–metal cations in a water-chloroform system revealed that the addition of MeCN in the organic phase led to a remarkable increase in sodium extraction relative to that in the absence of MeCN [101]. To explain these findings, we isolated two complexes from the organic phase of the extraction processes and determined their structures by X-ray diffraction. Crystals grown in the presence of MeCN showed the solvent molecule within the receptor’s hydrophobic cavity, while the cation occupied the hydrophilic cavity, coordinated by eight oxygen atoms (four ethereal and four carbonyl). Conversely, crystals obtained in the absence of MeCN exhibited a reduced coordination number of seven (four ethereal and three carbonyl oxygens), which accounts in part for the decreased stability of the complex in MeOH.

To further explain the role of acetonitrile in host–guest complexation involving calixarene derivatives, following thermodynamic studies to assess the medium effect on the complexation between lower-rim ketone calix[4]arene derivatives, **14** and the sodium cation in DMF, MeCN, and benzonitrile, PhCN, we isolated the Na.MeCN.calix[4]arene ketone complex. X-ray diffraction studies demonstrated that the sodium cation resided within the hydrophilic lower rim cavity, coordinated by eight oxygen atoms consisting of four ethereal and four carbonyls. Concurrently, a single MeCN molecule is again hosted in the hydrophobic pocket of the macrocycle [102,103]. The versatile coordination behavior of calix[4]arene derivatives was further investigated by comparing lower-rim ester and ketone derivatives, **12**, **13a** and **b**, **14** with bivalent cations in MeCN [104]. This resulted in the successful isolation of four distinct complexes, including two involving the ester derivative and two involving the ketone derivative, showing how altering the lower-rim functional group modification in the metal cation coordination dictates complex structure. Their structures, determined by X-ray crystallography, showed acetonitrile inclusion in their hydrophobic cavities.

The most remarkable feature of these structures and in our knowledge, at the time was unique in the solid-state calixarene chemistry is the role of the MeCN solvent in the Cd(II) complexes as a provider of a coordination site for the cation. For the Cd(II)-ester complex, the Cd(II) cation exhibited nine-coordinate geometry, binding to four ethereal and four carbonyl oxygens and the N atom of the MeCN solvent. In the Cd(II)-ketone complex, this changed to eight-coordinate geometry, where the cation binds to four ethereal oxygens, three carbonyl oxygens, and the MeCN nitrogen. This preference for soft N-donation highlights the soft character of Cd(II). Conversely, the Pb(II) complex exhibited an eight-fold coordination involving four ethereal and four carbonyl oxygens. This resulted in an unusual orientation of the MeCN molecule in the Cd(II) cavity, where the CN group pointed inward to form the Cd-N bond, which is inverted compared to the free ligand, the Pb(II) complexes, and others reported in the literature [104]. Furthermore, the lack of complexation of these cations and these ligands in MeOH, DMF, DMSO, and PhCN led to the conclusion that the calix[4].MeCN adducts rather than free ligands are responsible for the complexation of metal cations in acetonitrile.

Our work demonstrated that higher ketone basicity relative to esters increases complex stability. Cation desolvation and ligand binding thermodynamics revealed a Ca(II) selectivity peak in Gibbs energies, mirrored by enthalpies, except for Mg(II).

Incorporating a phenol unit in the cyclic tetramer to form calix[5]arene and its ester derivative, **15** led to (i) a loss of solvent selectivity with uniform transfer Gibbs energies. Additionally, (ii) ^1^H NMR showed unidentified alkali–metal active sites of interaction due to the isolated methylene bridge revealed in the ^1^H NMR chemical shifts, and the absence of cavity-bounded MeCN supported the ‘glove stretcher’ concept introduced by Danil de Namor, [105] where hydrophilic metal coordination opens the hydrophobic cavity for solvent penetration.

Regarding calix[4]arene tertiary amide derivatives, it is well established that their complex stabilities are stronger than those of ketones, which in turn are stronger than those for the esters [97]. The selectivity of tertiary amide calix[n]arenes (*n* = 4, 5, 6), **16a**, **16b**, **16c** toward metal cations in MeOH and MeCN was extensively discussed by Danil de Namor and colleagues in a 2013 review article [106]. We demonstrated that their binding ability decreased significantly when moving from the cyclic tetramer to the pentamer and disappeared almost entirely for the hexamer. Readers are also referred to research articles on calix[4]arene amide derivatives and alkali–metal cations published by Tomišić and colleagues [107,108,109].

Because calix[4]arene derivatives (esters, ketones, amides) contain oxygen donor atoms in their pendant arms, we investigated their interactions with trivalent lanthanide cations in dipolar aprotic solvents (DMF, MeCN) to evaluate conditions relevant to purification. The binding stability seems to be dictated by the basicity of the pendant arms‘carbonyl oxygens (amide ˃ ketone ˃ ester). The calix[4]ester, **12** interacts weakly while the ketone derivative, **14** binds cations but lacks selectivity due to a full enthalpy–entropy compensation effect. In contrast, the amide derivative, **16a** exhibited selective ion recogn ition in both media. Overall, complexation is a competitive equilibrium governed by both, cation desolvation and ligand binding. When the selectivity factor of one ion is plotted against the cation radii of lanthanides, a selectivity peak was obtained in both solvents, shifting depending on which of these two competing processes predominate [110].

#### 3.4.3. Calixarene Amines

##### Pyridino Calix[4]arenes

Building on the pioneering work by Pappalardo et al. [111,112,113,114,115] our research addressed the gaps in the solution and solid-state behavior of pyridinio calix[4]arenes. While previous studies reported poor extracting power of **17a** for alkali–metal cations in CDCl_3_ [114], we thought that this was due to the high degree of ion-pair formation in low-permittivity solvents rather than poor affinity of the receptor. To investigate these systems further, we conducted a detailed thermodynamic characterization of receptors **17a**, **17b** and **17c**, yielding relevant information on the following:

(i) Solution equilibria and structural properties. Thus, by determining the protonation constants of **17a** in MeOH [116], we successfully identified the optimal pH range to prevent proton interference during metal–cation binding. Conductance measurements confirmed 1:1 stoichiometries for alkali–metal and silver complexes of **17a**, in MeCN and PhCN, while ^1^H NMR studies in CD_3_CN demonstrated that complexation occurred via coordination with the phenolic oxygens and the pyridyl nitrogen. The ligand demonstrated high selectivity for the Li(I) cation over other alkali–metal cations, driven by favorable enthalpy and entropy contributions to the Gibbs energy of the process. The selectivity patterns hold in MeCN, where the ligand discriminated against Cs(I) in PhCN; a similar selectivity pattern is observed, but weaker complexes are formed. Furthermore, no complexation occurred with the larger Rb(I) and Cs(I) ions in PhCN.
(ii) The structural relationship between the monovalent cations and **17a** in solution is maintained in the solid state, as directly confirmed by the crystal structure of the 1:1 monoacetonitrile and silver complex (with perchlorate as the counter-ion) [117] as well as the Na.MeCN.**17a** complex. However, the X-ray superstructure of the Na.MeCN.**17a** revealed a structural feature that was entirely concealed during solution ^1^H NMR studies in CD_3_CN. Namely, the crystal lattice contains three distinct crystallographic complexes, two residing on a four-fold axis and one located on a two-fold axis [118]. In addition, two pyridino complexes in the solid state were isolated. These resulted from the direct displacement of chloride ions from AuCl_4_^−^ and PtCl_6_^2−^ in the coordination sphere of the central metal atom [119].
(iii) In our earlier studies, we established that the position of the pyridine nitrogen relative to the phenolic oxygen [116,117,118] significantly influences the basicity of the receptor; specifically, increasing the distance between these donor atoms enhances receptor basicity. While the parent macrocycle demonstrated negligible cation affinity, our investigation into the pyridino calix[4]arene derivative **16c** highlighted the considerable potential of modifying these binding sites for bivalent cations [120]. Through ^1^H NMR titrations in CD_3_CN, we demonstrated that the pyridyl nitrogen plays a primary role in complexing metal cations, such as Zn(II), Hg(II), Cd(II), and Pb(II). Conductance measurements further revealed the complexation of **17c** with Cu(II), Ni(II), Co(II) and Zn(II), with the formation of 1:1 complexes except for Hg(II), which forms a 2:1 (cation:ligand) complex. In the absence of suitable crystals for X-ray diffraction studies, molecular modeling suggested that each Hg(II) cation interacts with two adjacent N atoms, while MeCN occupied the hydrophobic cavity of **17c**. In MeOH, we found that **17c** complexed only with Hg(II), exhibiting a strong interaction, again yielding a 2:1 complex. A key focus of our research was comparing the complexation thermodynamics of **17c**, which is conformationally restricted, with its monomeric analog, **17d**, which can freely orient itself around the ion [120]. In acetonitrile, **17c** demonstrated a significantly higher hosting capacity and affinity for Hg(II) compared to other bivalent cations. Notably, the affinity of the 1:1 complex of **17c** for Hg(II) exceeded that of Cd(II), Cu(II), Ni(II), Pb(II), Co(II) and Zn(II) by factors ranging from 8.5 × 10^5^ to 83 × 10^6^. We determined that enthalpy control, accompanied by an entropy loss, was due to the conversion of two separate species (ligand and cation) into a single complex for all cations, except for Hg(II), whose complexation Gibbs energy values remained almost constant due to an enthalpy–entropy compensation effect. Conversely, the monomer **17d** in MeCN interacted with two receptor units to form a 1:2 (cation:ligand) complex, except for Pb(II). While **17d** maintained a higher selectivity for Hg(II) over other metal cations by factors of 155 to 398 compared to Cu(II), Pb(II), Zn(II), Ni(II), and Co(II), we observed a remarkable reduction in selectivity when moving from the macrocycle to the monomer. Finally, we concluded that while **17c** is highly selective for Hg(II), its application as an extracting agent for removing this cation from water is limited by the very high stability of the Hg(II) complex and the difficulty in regenerating the receptor. In this context, we found the monomer **17d** to be a more practical choice.
(iv) The statement made by Pappalardo and colleagues [114] regarding the relationship between the extraction of cations by pyridine calix[4]arenes and the kinetics of the complexation process was discussed [121] in light of the individual processes involved in the overall extraction of cations by neutral macrocycles as discussed by Danil de Namor and colleagues [121,122].

##### Acyclic and Aliphatic Amino Calixarenes

To expand the scope of calix[4]arene derivatives, our interest focused on the synthesis, structural and thermodynamic characterization and complexation of calix[4]arene tertiary amines, with the aim of targeting toxic metal cations and anions (when protonated) [123]. The X-ray structure of the diethylamine calix[4]arene, **18** showed a ‘cone’ conformation with a hydrophobic cavity enclosed by aromatic rings and a hydrophilic cavity containing phenolic oxygens and aminoethyl nitrogen donor atoms [124]. Solution thermodynamics in various solvents showed invariant transfer Gibbs energies from PhCN to other solvents due to an enthalpy–entropy compensation effect resulting from solvent reorganization [58]. In MeOH, acid-base properties revealed that each pendant arm behaves independently; speciation diagrams as a function of pH were reported prior to cation and anion complexation studies. Comparing the four protonation constants with those of fully functionalized pyridine calix[4]arenes, **17a**, **17b**, **17c** showed that the aliphatic amine calix[4]arene, **18** affinity for the proton is higher by factors of 10^15^ to 10^18^ [123]. For anion interactions, water–dichloromethane extraction with the protonated amine in the organic phase removed 84% of the gold(III)-containing anion of HAuCl_4_ from acidic aqueous media, outperforming the maximum of 59% [125]. The X-ray structure of an analogous complex with a piperidino-functionalized calix[4]arene, **19d** [125] explicitly confirmed direct interaction between the amine proton and the chloride atoms of the anion. For cations, ^1^H NMR studies disclosed that the cooperative effect of the four amino groups coordinated soft and toxic metal cations (Pb(II), Cd(II), Hg(II)) while discriminating against biological cations (Na(I), K(I), Ba(II)), which lacked access to the phenolic oxygen. These findings implied potential use for the removal of toxic metal cations from water, with the advantage of easy recycling via a pH-switching mechanism. Accordingly, the extraction abilities of seven calix[4]amine derivatives (three acyclic, three cyclic and one mixed pendant analog), **19a**–**g** for Ag(I) removal were investigated in water–nitrobenzene [126] and water–dichloromethane [127] solvent systems.

These studies highlighted the importance of establishing extraction stoichiometry. The process altered from the formation of a 1:1 complex in nitrobenzene to 1:2 (ligand:metal cation) in dichloromethane, changing the number of individual steps involved. Consequently, correlating ion–ligand stability in a single solvent with a phase-transfer extraction constant lacks validity. To eliminate the use of organic solvents, solid–liquid extraction was carried out, anchoring the macrocycle to a solid support, or using the receptor itself as a solid phase when water solubility was negligible. Examples include grafting two amino calix[4]arene derivatives (one fully and one partially substituted), **18**, **20**, onto silica for the removal of chlorophenoxy acid herbicides [128] and aspirin [129] from water. Attachment via the para position leaves the pendant arms free to interact with the guest *via* a proton transferred from the herbicide to the amino groups of the receptor, as confirmed by conductance measurements and solid-state X-ray diffraction crystallography [128]. Taking advantage of this proton affinity, a partially substituted solid-phase calix[4]arene bearing aminoethoxy pendant arms, **21** [130] was used for the selective extraction of pharmaceuticals commonly used (clofibric acid, diclofenac, aspirin) from water. This fundamental selectivity study addressed environmental pharmaceutical contamination. The solid receptor recognized these drugs selectively and discriminated other carboxylic drugs based on their acid dissociation constants (*pKa*), displaying a greater hosting capacity for clofibric acid and diclofenac (two protons taken up by the receptor) than for mono-protic alternatives.

#### 3.4.4. Calixarenes: Partially Substituted and Fully Substituted with Mixed Pendant Arms

The focus on the need to target Hg(II) cations due to their negative impact on human health and the environment led us to synthesize nine calix[4]arene derivatives [131] containing sulphanil groups in two of the pendant arms (partial functionalization) or fully functionalized by the incorporation of arms containing either a tertiary aliphatic amine or alicyclic or methyl thiophene or an amide functionality **22a**–**i**. At the time this research was carried out, phase transfer catalysts had been hardly explored except for the synthesis of calix[4]spherandiol [132], in which 18-crown-6, **7** was used to obtain the polyanion of the parent *p*-*tert*-butylcalix[4]arene, **11a** using tetrahydrofuran (THF) solvent and NaH as the source of complexation of 18-crown-6, **7** with the sodium cation. In our view, neither THF was the appropriate solvent to use (due to its low permittivity) for obtaining the naked calix[4] anion, nor was 18-crown-6, **7** selective for sodium. Consequently, acetonitrile was used as the solvent, and, considering the selectivity of 18-crown-6, **7** for potassium, a salt containing this cation was used, emphasizing the importance of considering solvent properties and catalyst–cation affinity to produce the reactive calix[4]arene anion.

X-ray diffraction studies of **22b** showed that in the solid state, this receptor presented a distorted ‘cone’ conformation as previously observed for other calix[4]arene derivatives, while conductance studies showed that **22b** interacted with Hg(II), Cd(II), Pb(II), Cu(II) and Ag(I). Further studies carried out with **22b** involved the medium effect on the complexation of this receptor with the Ag(I) cation [133]. Further studies involving **22j** involved a detailed investigation of the interaction of this ligand with metal cations in a variety of solvents relative to the calix[4]arene ester derivative, **12**. The latter contribution included ^1^H NMR and ^13^C NMR studies of **22j** in CDCl_3_, CD_3_CN, CD_3_OD and C_3_D_7_NO [134]. The findings suggested that MeCN resided in the hydrophobic cavity, implying higher selectivity for Hg(II) relative to other cations in this solvent. In fact, X-ray diffraction analysis revealed that recrystallizing the ligand in MeCN resulted in the solvent occupying the hydrophobic cavity of **22j**, whereas recrystallization from DMF showed the free ligand. Based on the stability constants of these cations with **22j**, a quantitative evaluation was made of how selectively this receptor binds Hg(II) compared with other cations. Notably, the selectivity for Hg(II) was 1778, 1862, 6918, 2434, 30,200 and 44,668 times higher relative to Pb(II), Na(I), Li(I), Cu(II), Ag(I), and Ca(II) in MeCN, respectively. The receptor displayed selective binding with Hg(II) and Ag(I) in DMF while remaining inert to any of the tested cations in PC. However, the selectivity for Hg(II) dropped significantly when switching from MeCN to DMF; in fact, in this solvent, the receptor is 2.6 times more selective for Hg(II) than for Ag(I). It was striking to find that in MeOH the selectivity reversed, with the receptor exhibiting a 10233-fold higher affinity for Ag(I) compared to Hg(II). The ligand exhibited discrimination against large alkali and alkaline-earth metal cations, while the interaction with the small ones must be *via* the oxygen donor atoms of the ester pendant arms, as disclosed by X-ray diffraction analysis of the sodium-MeCN-**22a** complex.

To prevent the removal of biologically essential ions, such as Na(I) and Ca(II), and to lower the stability constant of Hg(II) (facilitating receptor recycling in solid–liquid extraction), a partially functionalized calix[4]arene with two thio- functionalities (**22a**) was designed to avoid the ester group‘s participation [135]. Ligand–solvent interactions were analyzed by dissolving the ligand in CDCl_3,_ titrating it with various solvents (CD_2_Cl_2_, CD_3_OD, C_3_D_7_NO) and performing ^1^H NMR titration. Although a ‘cone’ conformation was suggested for the receptor in all solvents, higher deshielding of the aromatic protons indicated a ‘flattened cone’ conformation, a behavior prevalent in CD_3_CN. Complexation occurred exclusively with Hg(II) and Ag(I), while no interaction was observed with alkali, alkaline-earth, and transition metal cations. Thermodynamic analysis revealed that solvent effects on the host, guest, and complex [136] dominated the complexation, with the receptor showing selectivity for Hg(II) over Ag(I) in MeCN, which reversed in the alcohols and disappeared in DMF. **A paramount finding of this research was the introduction of a unique structural paradigm to the field of macrocyclic thermodynamics: the additive, individual contribution of pendant arms to the overall thermodynamics of calixarene complexation processes [137]. Through this framework, the overall thermodynamics of Hg(II) complexation with 22j in MeCN was firmly established using data for 22a and the calix[4]ester derivative containing common functionalities in the lower rim 12, representing a unique example of additive, individual arm contribution to the overall binding process, shown as follows. Based on the experimental thermodynamic parameters of complexation of Hg(II)12 (four equal pendant arms)**, (Δ*_c_G*° = −21.1 kJ/mol, Δ*_c_H*° = −21.1 kJ/mol, *T*Δ*_c_S*° = −0.1 kJ/mol), Hg(II)**22a** (two equal pendant arms. (Δ*_c_G*° = −37.2 kJ/mol, Δ*_c_H*° = −74 kJ/mol, *T*Δ*_c_S*° = −36.8 kJ/mol), Hg(II)**22j (Δ*_c_G*° = −49.6 kJ/mol, Δ*_c_H*° = −86 kJ/mol, *T*Δ*_c_S*° = −33.2 kJ/mol**). The contribution of each pendant for Hg(II)**12**, (Δ*_c_G*° = −21.1 kJ/mol/4 = −5.3 kJ/mol, Δ*_c_H*° = −21.1 kJ/mol/4 = −5.3 kJ/mol, *T*Δ*_c_S*° = −0.1 kJ/mol)/4 = 0 kJ/mol) and for Hg **22a** (Δ*_c_G*° = −37.2 kJ/mol/2 = −18.6 kJ/mol, Δ*_c_H*° = 74 kJ/mol/2 = −37 kJ/mol, *T*Δ*_c_S*° = −36.8 kJ/mol)/2 = −18.4 kJ/mol). Based on the contribution of individual pendant arms for Hg(II)**12** and Hg(II)**22a**, we calculate the thermodynamic data for Hg(II)**22j** containing two pendant arms common to Hg(II)**12** and two pendant arms common to Hg(II)**22a**. Thus, the thermodynamic parameters of complexation for Hg(II)**22j** are Δ*_c_G*° = 2 × −5.3 kJ/mol + 2 × −18.6 kJ/mol = −47.8 kJ/mol, Δ*_c_H*° = 2 × −5.3 kJ/mol + 2 × −37 kJ/mol = −84.6 kJ/mol, *T*Δ*_c_S*° = 2 × 0 kJ/mol + 2 × −18.4 kJ/mol = −36.8 kJ. These data are in good agreement with the thermodynamic data experimentally determined for Hg(II)**22j** shown above in bold letters.

A novel Hg(II) selective material was developed by immobilizing a partially functionalized calix[4]arene containing diethyl thiophosphate amino pendant arms, **24** onto a silica support. Based on detailed structural, electrochemical, and X-ray diffraction studies against a fully substituted calix[4]resorcinarene, **25** [138], this receptor created a highly recyclable material for the removal of Hg(II) from water. The immobilized matrix retained the original solution-phase selectivity for Hg(II) over Cu(II) and Ag(I), demonstrating how fundamental coordination studies directly inform the successful design of solid-state decontaminating agents.

One of the most promising biomedical applications of calixarenes lies in their development as novel anticancer agents. Extensive research has focused on this area, highlighted by the comprehensive reviews of Nimse and Kim [139] as well as Daun and colleagues [140], which underscore the low in vitro toxicity of these macrocycles. However, despite the efficiency of various calixarene derivatives against diverse types of cancer, a significant knowledge gap existed regarding their applications against colorectal cancer, a disease of global concern due to its high annual mortality rate. To address this issue, we previously explored the structure design and thermodynamic evaluation of a partially functionalized calix[4]arene containing thioamide moieties, **26** on its pendant arms [141]. This receptor successfully inhibited Caco-2 cell proliferation in a concentration-dependent manner *via* the induction of apoptosis. MTT assays confirmed high potency against Caco-2 cells, demonstrating faster kinetics than standard chemotherapeutic drugs, while flow cytometry validated the onset of the apoptotic cascade at 10 µM. Furthermore, ^1^H NMR and Raman spectroscopy corroborated these mechanisms, revealing altered metabolic profiles with depleted water-soluble metabolites and membrane phospholipids [142]. The Raman data proved that this calixarene receptor interacted with protein metabolites, modulating transmembrane transporters to successfully bypass drug efflux-mediated resistance mechanisms.

#### 3.4.5. Ditopic Calix[4]arene Derivatives

Although typically known for binding cations, incorporating suitable functional groups in calix[4]arenes led us to enhance their ability to act as cation/anion binders or ditopic receptors. With this in mind, we have modified these structures by synthesizing a partially substituted narrow-rim calix[4]arene hydroxyamide derivative, **27** [143]. Its capacity to interact with cations and anions was evaluated in MeCN, MeOH and DMSO. Ligand–solvent interaction was found in MeOH and DMSO. The receptor was able to recognize alkaline-earth, zinc, and lead metal cations. A plot of Gibbs energies of complexation in MeCN versus cation solvation revealed a selectivity peak for Mg(II), highlighting that the balance between desolvation and binding drives the receptor’s metal cation selectivity. Furthermore, investigation of anion interactions with fluoride, dihydrogen phosphate, and pyrophosphate through hydrogen bond formation in MeCN and DMF demonstrated that the receptor functions as a ditopic host in dipolar aprotic media.

#### 3.4.6. Calix[4]arene Derivative for Anion Complexation

While our previous work focused on lower-rim-functionalized calix[4]arenes, our recent investigations have shifted to wider-rim calix[4]arene derivatives incorporating azo functionalities. The design of chromophores with multifunctional sensor systems has attracted significant attention for their utility in chemical sensing. These sensors exhibit naked-eye color changes upon interacting with ions or molecular species, bypassing the need for complex spectroscopic techniques. Particularly, azophenol-based chromophores display both altered absorption spectra and visible color changes when interacting with metal cations or anions. The incorporation of azo functionalities has been the subject of interesting review articles by Suksai and Tuntulani [144] and Dutta and Sahana [145]. While the main interest in supramolecular chemistry relates to thermodynamics, little attention has been directed toward the resulting salts and their applications.

To our knowledge, there are few prior reports regarding CO_2_ uptake by anion-complex salts of calix[4]arenes in both solution and atmospheric phases. To address this, we investigated a highly recyclable chemosensor probe designed for detecting and capturing CO_2_, which was based on the fluoride complex salt of *p*-ester diazo-phenyl calix[4]arene, Bu_4_N (CA-AZ), **28** [146]. Our work involved fundamental studies on the synthesis, characterization, and properties of this free receptor. Based on our findings, we concluded that (i) the receptor underwent conformational changes as a result of the medium effect and upon complexation, as confirmed by ^1^H NMR analyses in DMSO-d_6_; (ii) complexation thermodynamics demonstrated that the receptor is highly selective for the fluoride anion relative to other 1:1 complexes; (iii) the interaction between the anion and receptor is initiated by the deprotonation of a single phenolic unit located in the narrow rim of the receptor followed by a subsequent protonation at the upper rim, which generated the active coordination site for the target anion; and (iv) The anion-complex salt demonstrated a robust capability to capture and remove CO_2_ from the air. The complex salt can be easily recycled, making the entire system reusable. This chemosensor offered several advantages, including straightforward synthesis and fast reaction kinetics. Furthermore, the chemical interaction with CO_2_ induced a colorimetric shift that allowed visual detection by the naked eye without the need for analytical instruments.

### 3.5. Calixpyrroles

Calixpyrroles is the name given by Sessler and colleagues [147] to these receptors, products of the reaction between pyrrole and ketones in acidic medium. The origins of calixpyrroles can be traced back to 1886 with the initial synthesis by Baeyer [148], followed by several subsequent contributions [149,150,151]. However, the significance of these macrocycles as anion receptors was primarily established through the excellent work of Sessler, Gale, and colleagues in the 1990s [152,153,154,155,156].

While cation receptors are numerous within the area of supramolecular chemistry, research into anion receptors has been limited; consequently, the development of anion receptors has generated a great deal of excitement in the scientific community. Interest in calixpyrroles stems from their straightforward synthesis and the number of sites available for functionalization.

#### 3.5.1. Calix[4]pyrrole Complexation with Anions

Although Sessler and Gale pioneered much of the work in this area, some of the data reported on the stability constants for parent calix[4]pyrrole–halide, **29** complexes required re-evaluation, as reports in the literature questioned existing data in MeCN [157]. To address this controversy, we performed a comprehensive thermodynamic analysis in MeCN and DMF [158], evaluating every step (Scheme 1) in terms of Gibbs, energies, enthalpies and entropies to ensure that the data accurately represented the processes taking place in solution. Studies concluded that the parent calix[4]pyrrole, **29** exhibits selective halide recognition in both solvents, with binding energies driven by charge density. The ligand successfully competes with the solvent for the anion, resulting in stronger hydrogen bonding between the host and the higher-charge-density fluoride anion.

Then, we aimed to explore how calix[4]pyrrole selects non-spherical anions, testing a diverse range including phosphate and arsenic species. We found it particularly interesting that the receptor engaged with biologically and environmentally relevant phosphate and arsenic species. Consequently, we applied the same analytical approach used for spherical anions to these specific targets in MeCN and DMF. Regarding phosphates [159], calix[4]pyrrole, **29** interacted with dihydrogen phosphate in a 1:1 stoichiometry in MeCN and DMF. While the binding is enthalpically driven with an entropic loss in MeCN, both enthalpy and entropy contribute favorably to the Gibbs energy in DMF. Conversely, hydrogen pyrophosphate binds with two receptor units in both solvents, and these processes are driven by enthalpy. An enthalpy–entropy compensation effect explains the similar stabilities observed for both anions across both solvents.

Regarding arsenic, our focus was investigating the interaction between calix[4]pyrrole, **29** and arsenic species to develop a selective receptor for removing arsenic from water [160]. Funded by the EU, this project addressed the lack of available technology for treating arsenic-contaminated water in partner regions, specifically Argentina, Brazil, and Perú. Based on ^1^H NMR and simulation results showing binding with both As(III) and As(V), solid–liquid extraction studies were conducted. These experiments optimized key parameters including material mass, pH, temperature, interferences, and kinetics to determine the maximum efficiency of the material for arsenic removal. Calix[4]pyrrole demonstrated fast extraction kinetics, removing 15.28 mg/g and 14.29 mg/g of As(V) and As(III), respectively. Tested on real contaminated samples from Argentina (San Cristóbal, Santa Fe Province, and Jacal River, San Juan Province), the material achieved over 85% removal of As species from contaminated water. This ability to tackle arsenic in environmental samples positions this material for its transition from successful laboratory testing to large-scale technological integration.

#### 3.5.2. Anion Complexation by Calix[4]pyrrole Derivatives

Although fluoride helps to control tooth decay, in excess, it can damage soft tissues and add complexity to tooth development. Also, phosphates are known to be nutrients; they can be toxic when combined with fluoride in industrial environments. Consequently, this study explored phosphate and fluoride recognition using (i) a double cavity calix[4]pyrrole derivative, **30** [161], (ii) two calix[4]pyrroles isomers, **31a**, **31b** [162], and (iii) a structural modification of calix[4]pyrrole. These approaches are summarized as follows:

(i) Macrocycles featuring two cavities or semi-cavities offer higher guest-hosting capabilities compared to their single-cavity counterparts. This study focused on a double-cavity calix[4]pyrrole derivative, **30** designed to bind two fluoride anions per ligand unit. ^1^H NMR studies were striking, revealing both the active sites of interaction of the ligand with the anion and the sequence of events in the binding process. The results indicated that the initial hydrogen bond formation between the pyrrolic hydrogen of the macrocycle and the anion is followed by the uptake of a second fluoride anion with the phenyl urea moiety. Both complexation steps are enthalpically and entropically favored. The increase in entropy in both steps was attributed to the desolvation of the anion upon complexation. Beyond interacting with fluoride, this receptor enters complexation with dihydrogen phosphate using hydrogen bonds *via* its phenyl urea moieties while discriminating against other halides, hydrogen sulfate, perchlorate, nitrate, and triflate anions. There is scope for further work aiming to immobilize the receptor by crosslinking its phenyl groups with formaldehyde in an acidic medium to produce a polymer.
(ii) It was fascinating to discover that the reaction between pyrrole, 3-hydroxyacetophenone, and methane sulphonic acid in methanol yielded two distinct isomers (αβαβ and ααββ), **31a**, **31b** upon several recrystallizations in different media. Conclusions derived from ^1^H NMR, conductance measurements, and thermodynamic analysis (Scheme 1) indicated that these isomers exhibited unique anion-binding properties: ααββ, **31b** formed 1:2 (ligand:anion) complexes with dihydrogen phosphate but only 1:1 complexes with other anions (including fluoride) in MeCN, while αβαβ, **31a** formed 1:2 complexes exclusively with fluoride in DMF. However, in moving from DMF to MeCN, the latter can take up two hydrogen phosphate units as well as two fluoride units per unit of receptor. This reflects the inherent nature of the solvent and its significant role in the complexation process.
(iii) To tune the anion recognition properties of calix[4]pyrrole by designing and synthesizing a thiophene-modified derivative, **32** [163]. This research aimed to analyze the effect of the heteroatom insertion on anion-binding strength, with a special focus on assessing how the properties of the solvents (DMF, DMSO, MeCN, PC) impacted the host–anion complexation process. A distinctive characteristic of this receptor lies in its Gibbs energy of transfer from MeCN to other solvents. Unlike calix[4]pyrrole, this receptor exhibits a solvation selectivity sequence DMF ˃ DMSO ˃ MeCN ˃ PC. ^1^H NMR data in deuterated MeCN confirms that anion complexation occurs via the pyrrolic NH hydrogen bonding. Replacing a pyrrole unit with thiophene significantly diminished the chemical shift response, as the changes observed between calix[4]pyrrole and the modified receptor underscore the significant impact of replacing a pyrrole unit with thiophene on the strength of complexation. In this medium, the receptor forms a 1:1 complex with spherical halides and a 2:1 (ligand:anion) stoichiometry with tetrahedral dihydrogen phosphate. While fluoride selectivity is shown across all solvents, the stability of these complexes varies significantly, as shown in this study, based on the differential solvation between reactants and the product. In PC, stabilities are lower than in MeCN, and the ligand–dihydrogen phosphate composition shifts to 1:1. As the basicity of the solvent increases as in DMF and DMSO, complexation becomes increasingly limited to a few ions. Much like calixarenes, calix[4] pyrrole interactions observed in one solvent are not representative of those found in another solvent.

#### 3.5.3. Ditopic Calix[4]pyrrole Based Receptors

Building on our previous work with calixarenes, we have focused significant attention on ditopic receptors based on calix[4]pyrrole derivatives. These systems are highly relevant due to their capacity to accommodate two distinctive guests of environmental concern (heteroditopic). Our contributions to this field are outlined below:

(i) A new calix[4]pyrrole derivative, **33** was designed by replacing a methyl group on the parent calix[4]pyrrole with a sulfur-containing pendant arm [164], enabling interaction with soft metal cations like Hg(II) and Ag(I). Like the monomeric units of thiocalix[4]arenes, this structure eliminates the methylene bridges in calix[4]arenes, providing greater flexibility for the pyrrolic NH groups to engage with anions. The resulting structure featured four sulfur-containing pendant arms, creating room for accommodating two Hg(II) metal cations. These statements have been corroborated by ^1^H NMR, conductance, and thermodynamics. The remarkable feature of this research is that this modified calix[4]pyrrole derivative effectively discriminates against anions, except fluoride, and hosts two mercury(II) cations, avoiding the interference from biologically essential ions. Such characteristics are promising for further research at pilot-plant scale with the aim of developing a technological approach for removing these environmental pollutants.
(ii) Building on the above, we synthesized a modified calix[4]pyrrole by substituting a meso-methyl group with a phenoxy methyl ketone moiety, **34** [165]. Subsequently, we characterized the structure and thermodynamics of its binding, including corresponding solution studies on the reactants and the product participating in the process. ^1^H NMR studies demonstrated that this ligand interacts with F^−^ in CD_3_CN. Among the cations (alkali, alkaline earth, transition and heavy metals), chemical shift changes were only observed with Hg(II).

Strikingly, this derivative exhibited better selectivity compared with the thiocalix[4]pyrrole, **33;** while the latter binds both Hg(II) and Ag(I), the present receptor interacts only with Hg(II) through the oxygen atoms of the pendant arms. Conductometric titrations established a 1:1 binding stoichiometry for both the fluoride and mercury complexes. Furthermore, the thermodynamic profile across diverse media (MeCN, PC and DMSO) revealed very similar stability constants resulting from a pronounced enthalpy–entropy compensation effect. Building on these results, the base-catalyzed phenol–formaldehyde method developed by Blasius and colleagues [166] was used to synthesize the oligomer. This approach was then optimized for the extraction of fluoride and mercury from water across various experimental conditions. Remarkable capacities for fluoride (214 mg/g) and mercury(II) (118.55 mg/g) were achieved, as assessed from reviews in this area [167,168]. The material was recycled via a pH-switching mechanism. These findings provide a foundation for scalable engineering applications with the aim of generating a water treatment technology targeting these contaminants. A homologous calix[4]pyrrole ketone derivative, **35** was synthesized and subjected to fundamental analysis [169]. A primary objective of this work was to clarify and correct misleading statements in the literature concerning the optimal choice of reaction media for macrocycle complexation processes

(iii) The application of calix[4]pyrrole derivatives in cation monitoring systems at the time this research was carried out remain underdeveloped. Consequently, fundamental studies to establish the host–guest thermodynamics were imperative to guide the rational selection of the ionophore for the development of ion-selective electrodes (ISEs) [170]. Addressing this, a calix[4]pyrrole derivative, **36** substituted with amide functionalities (designed to possess combined cation and anion recognition sites) was synthesized and characterized using ^1^H NMR and ^13^C NMR spectroscopy in CD_3_CN. This bifunctional receptor demonstrated competitive binding interactions with various metal cations: (Ca(II), Sr(II), Ba(II), Pb(II), Cd(II), and Hg(II). A 1:1 stoichiometry was observed with most cations, except Hg(II), which formed a 1:2 (ligand:metal cation) complex. These fundamental studies provided the foundation for designing a Hg(II) ion-selective electrode. Furthermore, a detailed investigation was conducted to assess interactions between the membrane components and the analyte in solution. A membrane prepared without the receptor revealed that the analyte interacts with the NaPh_4_B additive, showing Nernstian behavior. This interaction was further confirmed by ^1^H NMR spectroscopy. Furthermore, the selection of the plasticizer significantly influenced the electrode’s response. Surface analysis of the membrane was characterized by SEM-EDAX; the sensor‘s performance was evaluated by optimizing the pH, soaking time, response time, and reversibility, while also determining the lifetime of the electrode and selectivity coefficients. The sensor was successfully used as an indicator electrode for the complexometric titration of Hg(II) with EDTA and the quantification of Hg(II) in a dental amalgam. A comparison with existing literature showed advantages. In addition, the amide calix[4]pyrrole derivative **36** was used for the synthesis of a selective and easily recyclable dimer for the removal of mercury(II) from water [171]. The dimer was characterized by mass and FT-IR spectrometry, Scanning Electron Microscopy (SEM), and Energy-Dispersive Atomic X-Ray (EDAX). An interesting feature of the micrographs of the loaded dimer was the morphology change with the cation. The removal of mercury(II) from water was assessed under different experimental conditions to find the optimal extraction conditions for the removal of the toxic metal from water. The dimer was easily recycled *via* a pH-switching mechanism.

Selecting the ionophore is a critical step when developing ion-selective electrodes based on supramolecular chemistry. A key question is whether this choice should rely on selectivity or receptor-hosting capacity. To explore this, we compared two receptors: the calix[4]pyrrole derivative discussed above, **36** and a calix[4]arene derivative, **37** containing mixed amide and ketone pendant arms. In contrast with the latter, which forms 1:1 complexes, the former binds two mercury(II) cations per unit of receptor. Based on fundamental studies of these receptors, the calix[4]arene derivative, **37** showed a higher affinity for Hg(II) than the calix[4]pyrrole derivative, **36** in the formation of 1:1 complexes as assessed from their stability constants and the contributions of the enthalpies and entropies to the Gibbs energies of these processes, providing a quantitative protocol for selecting the receptor for ion-selective electrodes [172]. Potentiometric measurements revealed that both electrodes exhibited Nernstian behavior. However, the calix[4]arene derivative, **37** demonstrated better performance, characterized by higher selectivity for the 1:1 Hg(II) host–guest complex, a broader linear concentration range, and a faster response time. Due to the fast response time of the electrode, a 2:1 (metal cation:ligand) complex is not formed. Consequently, the increased hosting capacity of the calix[4]pyrrole derivative appears to have no effect on the overall electrode response. The enhanced sensitivity of the calix[4] arene-based receptor may be attributed to the ‘allosteric effect ‘ previously mentioned in the calix[4]arene framework, driven by the presence of MeCN in its hydrophobic cavity-a behavior not observed in calix[4]pyrrole. However, the most significant finding of this study is that the selectivity coefficient of a receptor for a given cation over another can be accurately predicted from thermodynamic stability constants. (Equation (12)):

log *Ks* M(II) − log *Ks* (Hg(II) = log *k* Hg(II)/M(II) (12)

Therefore, the selectivity coefficients *k* can be obtained without having to build an electrode. This relationship was further corroborated using a fully substituted calix[4]pyrrole derivative containing thioamide functionalities, **38** as pendant arms. The results obtained were compared with those of an earlier study of the partially substituted receptor containing the same pendant arms, **26**. While the fully substituted receptor binds two cations per unit of receptor, the rapid electrode response suggested that this stoichiometry was highly unlikely to take place in this system. Based on these investigations, two ISEs were successfully built [173]. Both electrodes demonstrated Nernstian behavior, although the detection limit for the electrode containing the fully substituted calix[4]pyrrole yielded a marginally lower detection limit (3.16 × 10^−7^ mol.dm^−3^) and a wider linear range than the electrode loaded with the partially substituted receptor. Both electrodes displayed similar response times (11 and 10 s). Equation (11) tested for the fully substituted receptor remained valid. Equation (12) could not be applied to the electrode with the partially substituted receptor because it interacts significantly with Hg(II), moderately with Ag(I), and negligibly with other cations. It was concluded that thermodynamics is a suitable reporter for the design of Hg(II) ISEs when MeCN is the medium.

#### 3.5.4. The Structural Diversity of Calix[4]pyrroles Allowed for Varied Functionalization

A novel, asymmetric calix[4]pyrrole derivative [174], N-rim, partially substituted calix[4]pyrrole derivative, **39**, featuring thioamide functionalities at the N-rim, was synthesized and fully characterized. The receptor‘s geometry makes it selective for Ag(I), while eliminating the ability to bind anions. While the variety of techniques used confirmed relatively strong interaction with Ag(I), attempts to bind Hg(II) resulted in structural destruction of the receptor. Molecular simulations suggested significant strain in bonds and angles of the receptor, leading to its destruction. Given the environmental toxicity of Ag(I) in aquatic systems, the synthesized material showed potential for removing silver from aquatic systems or to use it to produce an Ag(I) ion-selective electrode for in situ monitoring purposes.

Another area of interest was to explore sulfur-containing hetero-calix[4]pyrroles, **40a**–**c** as mercury(II) receptors [175]. These modifications included replacing one or two pyrrole units with thiophene units and an N-methyl derivative by substituting the pyrrole ring hydrogen with methyl groups or positioning thiophene at the meso site.

Through partition studies in the acetonitrile–hexane solvent system, it was verified that monomeric species of the receptor predominated at the tested concentrations. The mutual solubility of the solvents involved was also assessed by comparing data from partition (solvents mutually saturated) with those involving the pure solvents (transfer). Interaction of all receptors with Hg(II) was detected by ^1^H NMR, which revealed substantial conformational changes. No such interaction was observed with the parent calix[4]pyrrole, **29**. Conductance, UV-VIS spectrophotometry, and titration calorimetry revealed that all receptors formed 1:1 complexes with the exception of the mono-thiophene derivative. In conclusion, by incorporating soft donor atoms, thiophene-hybridized calix[4]pyrroles achieved highly selective binding with mercury(II) cations in MeCN. While complexation is enthalpy driven, the N-methyl derivative is governed by both enthalpy and entropy. Furthermore, the thiophene calix[4]pyrrole can be tailored via a basic-medium condensation reaction, unlocking sensing and Hg(II) decontaminating capabilities.

### 3.6. Calix[4]resorcinarenes

Calix[4]arenes are synthesized from *p*-substituted phenol and formaldehyde in basic medium, whereas calix[4]resorcinarenes are products *via* the acid-catalyzed condensation of resorcinol and aldehydes. The presence of eight phenolic hydroxyl groups, rather than four, significantly expands the potential for creating a wide variety of derivatives. The chemistry of calix[4]resorcinarenes, including the pioneering research that led to the synthesis of these macrocycles, is extensively documented in the chemical literature [176,177].

A summary of our contributions involved

(i) A detailed structural and thermodynamic study involving an ethyl thiomethylated calix[4]resorcinarene derivative, **41** and metal cations [178] in MeCN and MeOH. The most striking feature of this study is the versatile complexation behavior of this receptor with metal cations. Thus, following partition experiments (which demonstrated that the receptor remained in a monomeric state), studies were carried out with metal cations in MeCN and MeOH. This investigation showed that Hg(II) cations in MeCN and MeOH in excess of the ligand caused an increase in conductance, indicating complex decomposition and the formation of highly mobile, multicharged polymeric species, consistent with the X-ray structure of the isolated complex. Interaction with Cu(II) in MeCN led to the formation of metalates due to the poor solvation of this cation with this solvent. In contrast, MeOH yielded a 1:1 complex where highly favorable entropy and a low enthalpic stability indicated strong desolvation upon complexation. Unlike Cu(II), Ag(I) is well solvated in MeCN, and therefore, it was reluctant to complex with the receptor. Significant changes in the solvation of reactants and product resulted in higher stability and hosting ability (1:2 ligand:metal cation) in MeOH. It has been universally observed that a solvent that effectively solvates the reactants provides a poor medium for complexation.
(ii) Given that the fully substituted calix[4]resorcinarene derivative, **41** [179] underwent degradation and failed to achieve coordination with Hg(II) in both acetonitrile and methanol, a partially substituted analogous featuring identical pendant arms, **42** was targeted for synthesis. Subsequent structural and thermodynamic investigations demonstrated that this modification stabilized the system, yielding a new receptor that can interact with Ag(I) to give a 1:1 complex in MeOH. However, the hosting capability of the receptor for Hg(II) was significantly increased, binding two cations per receptor unit in MeOH, MeCN, and DMF. Conversely, in PC, the complex stoichiometry shifted, resulting in a 1:1 complex. Furthermore, unlike the fully functionalized macrocycle, partial functionalization of the framework led to the formation of stable Hg(II) complexes.
(iii) This investigation explores the synthesis, structural and thermodynamic properties of a calix[4]resorcinarene amide derivative, **43** interacting with cations [180]. To assess potential applications, thermodynamic data were used to quantify receptor selectivity through the ratio of stability constants. This information is critical for extraction processes where receptor selectivity dictates how well a specific guest was targeted. Due to the selectivity of this receptor for Ca(II) in the solvents investigated, investigations into optimizing the removal of Ca(II) from water were carried out, revealing a remarkable receptor capacity of 962 mg/g, significantly outperforming current standard commercial treatments.

## 4. Conclusions

Our fifty-year retrospective aims to demonstrate that while thermodynamics remains a fundamental science, its complex mathematical formalism becomes far more accessible when derived directly from experimental data. Prioritizing this application-first approach to reach formal theory will effectively enhances the accessibility of thermodynamics to a much wider audience, inspiring future generations of researchers to expand the frontiers of this field.

We advanced the thermodynamics of supramolecular chemistry by bridging fundamental binding studies with solution chemistry of the species participating in the process of complexation. This powerful combination sparked practical innovations, such as water remediation, the derivation of selectivity coefficients from thermodynamic data for the design of ion-selective electrodes, and the additive contribution of pendant arms to the thermodynamics of complexation, while providing the accurate enthalpy data needed to master the counter-ion effect on the coordination process and to check the reliability of enthalpy data.

While supramolecular chemistry traditionally focuses on complexation, the vast number of novel electrolytes generated by interacting neutral receptors with common salts remains understudied. The conversion of simple ions into complex ions and counterions yields new materials with promising applications. For instance, lithium coronand salts exhibited higher conductivity than conventional lithium common salts in battery-relevant solvents, while a fluoride-complexed azo-calix[4]arene efficiently captured ambient carbon dioxide. Consequently, further research is required in this emerging field.

While research has successfully proven the efficacy of these calix-based receptors at the laboratory scale, transitioning them to pilot-plant scale represents the next step toward full commercialization. Given their straightforward synthesis, exceptional selectivity, fast extraction kinetics, and regeneration mostly by a pH-switching mechanism, this remains an appealing and fertile area of research. It is our hope that future investigators, including ourselves, seize this opportunity to continue this research and proceed with pilot-scale trials, unlocking the impact of these materials for the development of new technologies for water purification.

## Figures and Tables

**Table 1:** Chemical structures of the macrocyclic receptors and their derivatives discussed in this review. The collection includes cryptands, crown ethers, cyclodextrins, calixarenes, functionalized calixarenes, functionalized calixresorcinarenes, calix[4]pyrrole and functionalized calix[4]pyrroles, illustrating the structural diversity and chemical modifications. The compounds are organized according to their structural class, and numbering corresponds to that used throughout the text.

No.	Structure	No.	Structure
**1a**	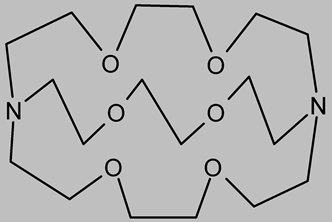	**1b**	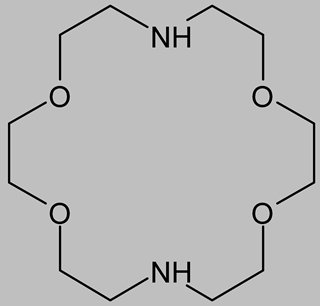
**2a**	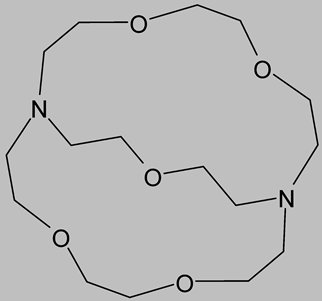	**2b**	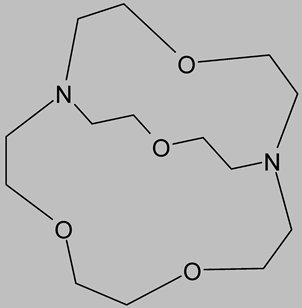
**3**	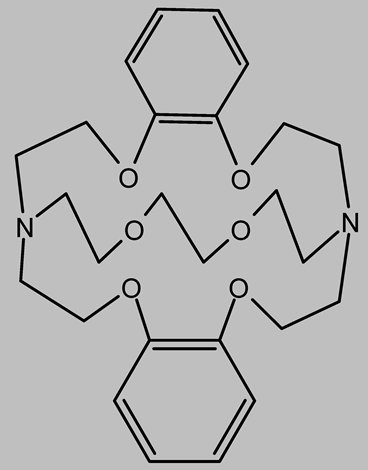	**4**	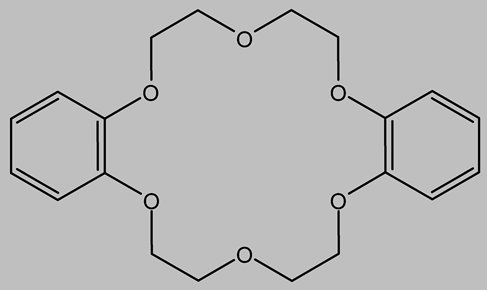
**5**	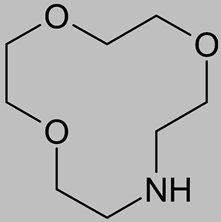	**6**	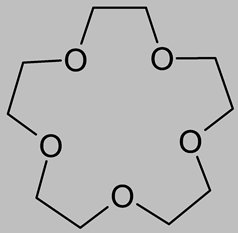
**7**	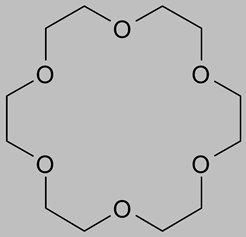	**8**	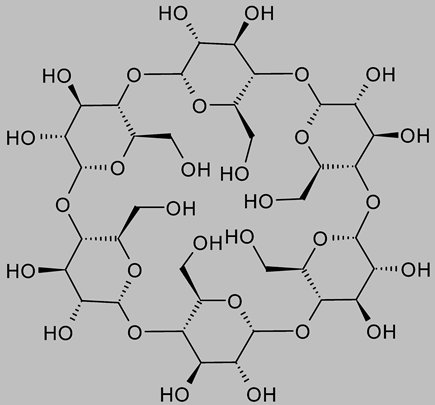
**9**	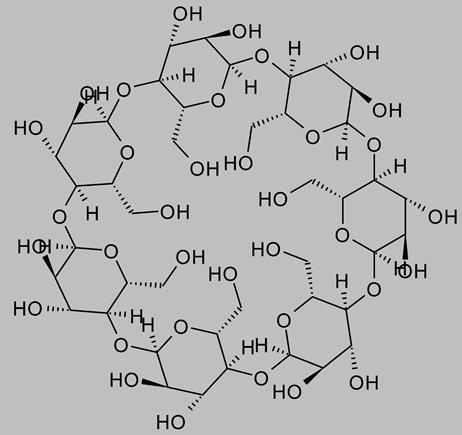	**10**	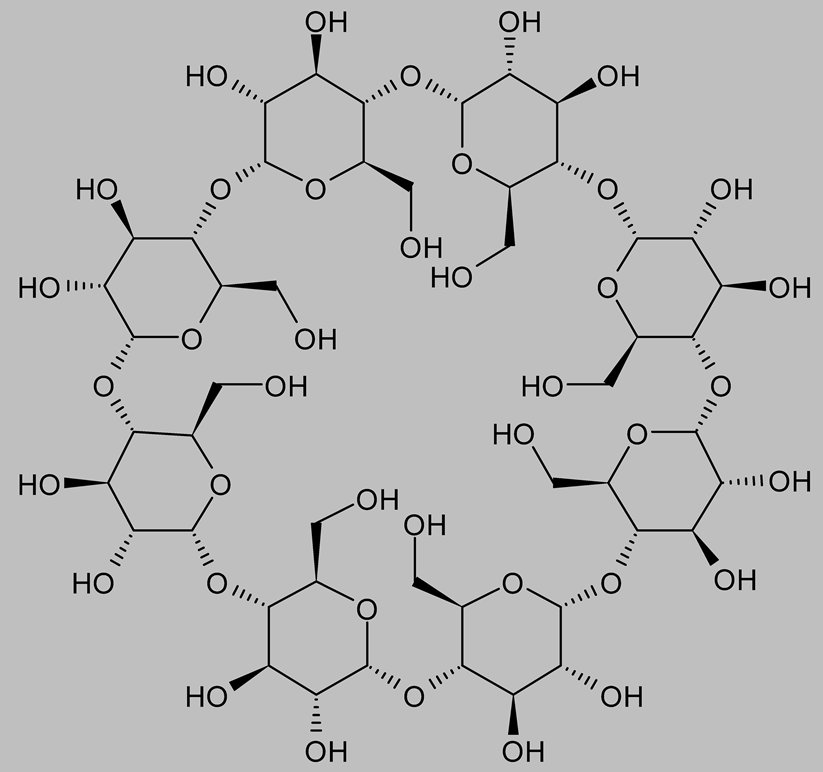
**11a**	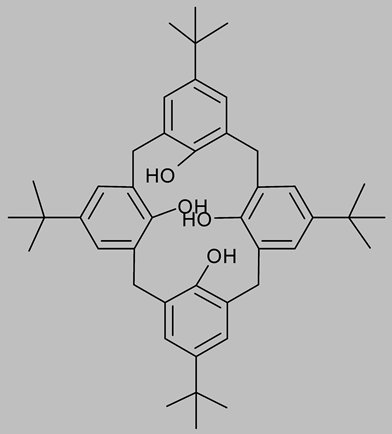	**11b**	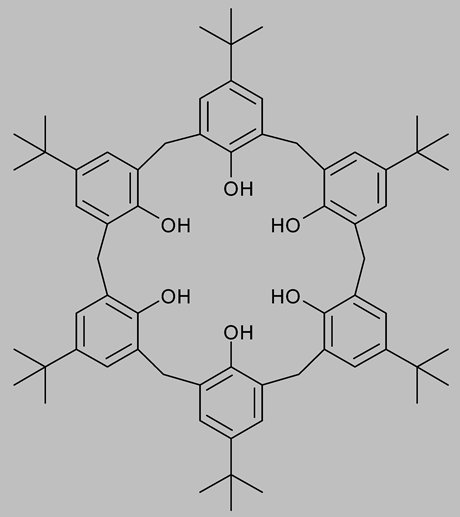
**11c**	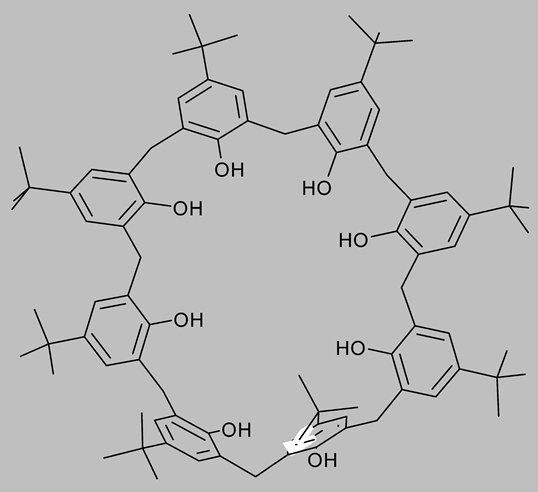	**12**	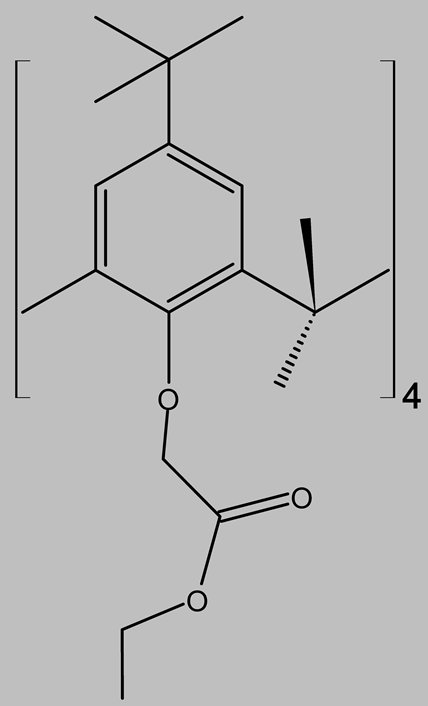
**13a–b**	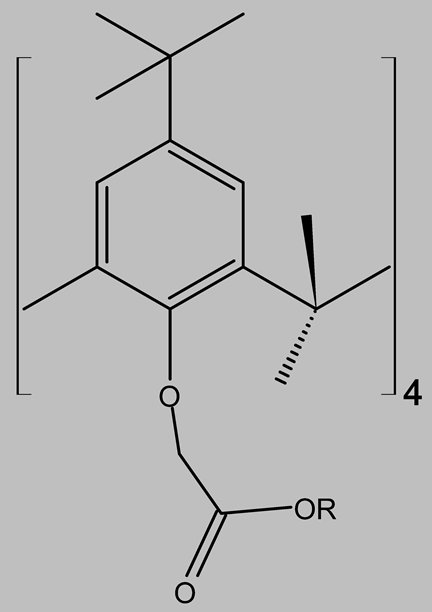 R = Methyl,13a Butyl,13b	**14**	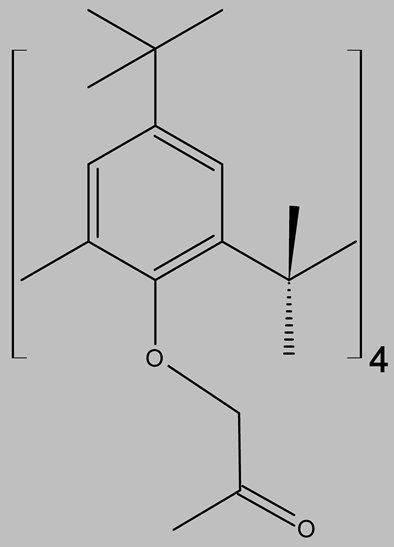
**15**	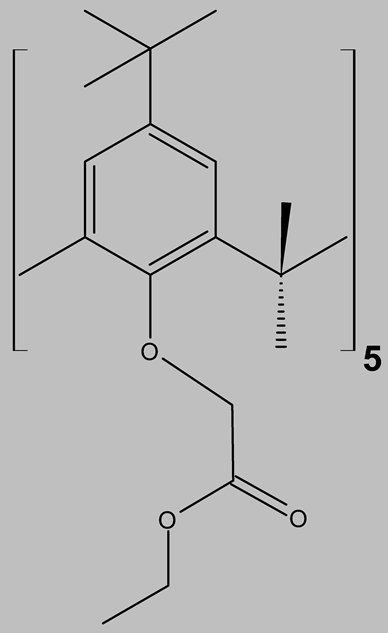	**16a**	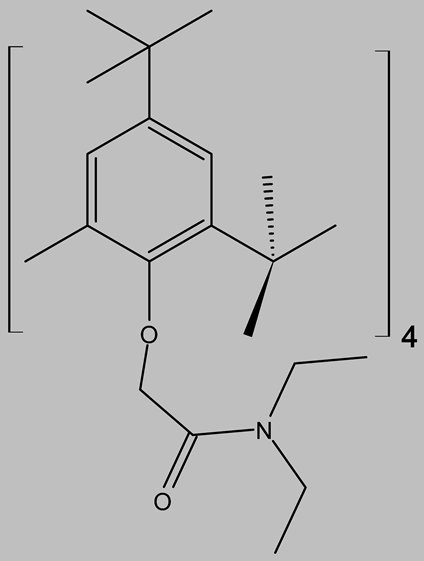
**16b**	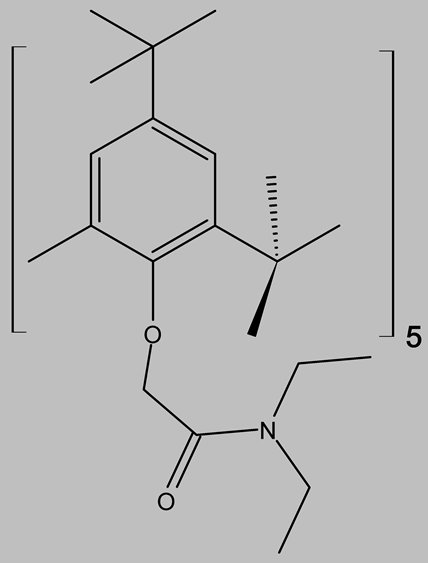	**16c**	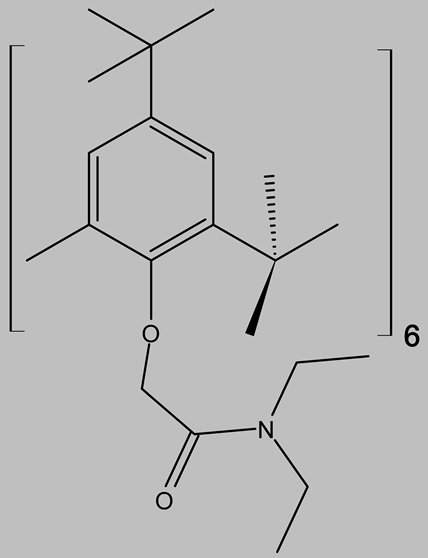
**17a**	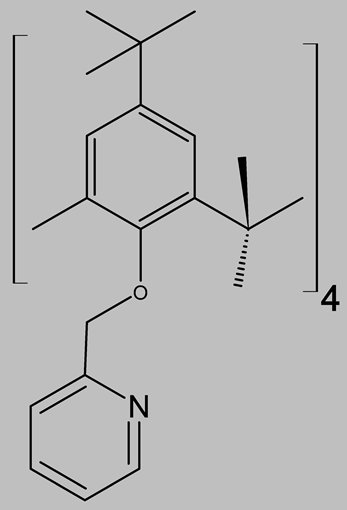	**17b**	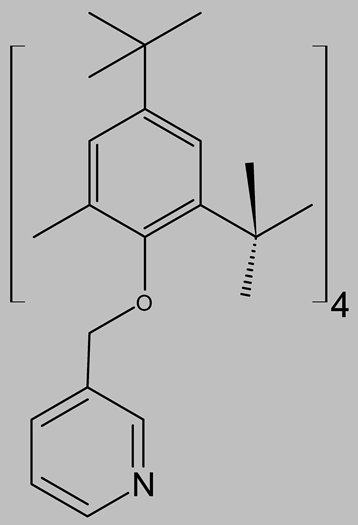
**17c**	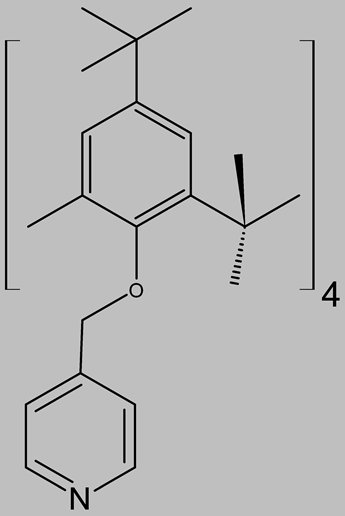	**17d**	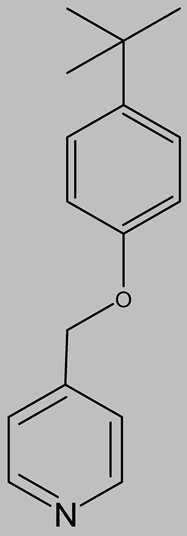
**18**	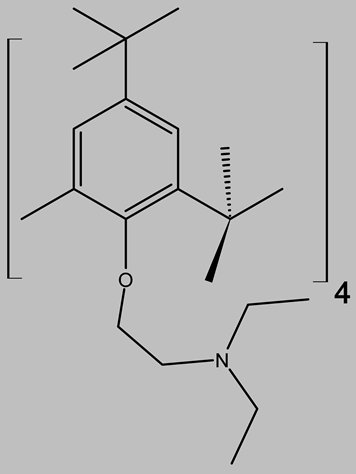	**19a**	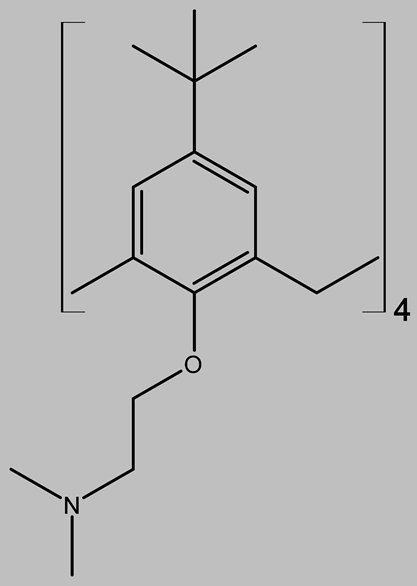
**19b**	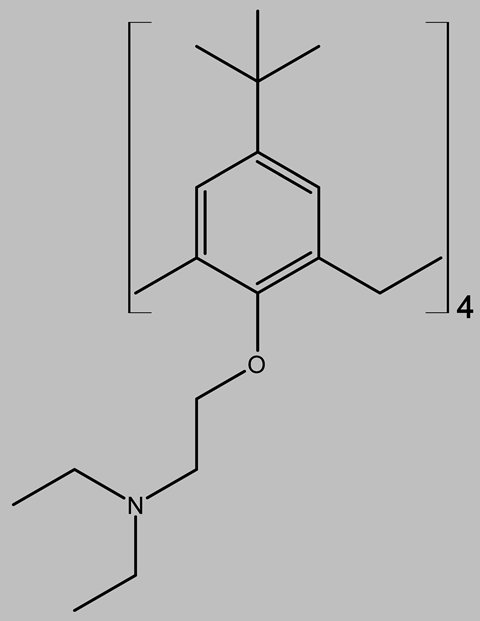	**19c**	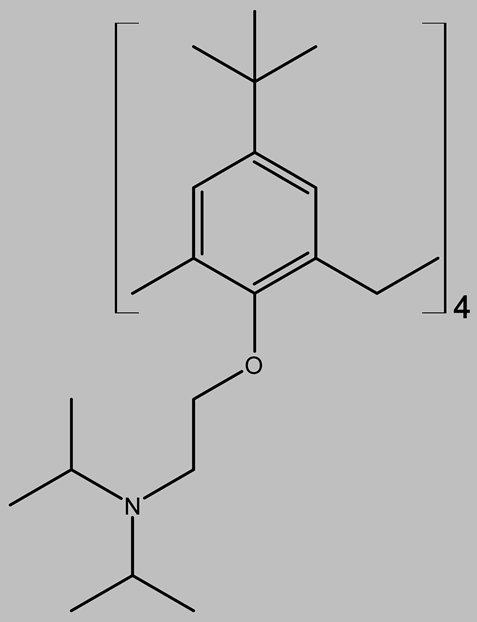
**19d**	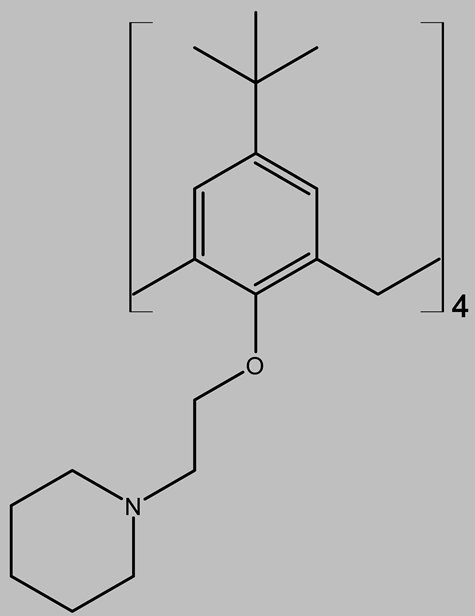	**19e**	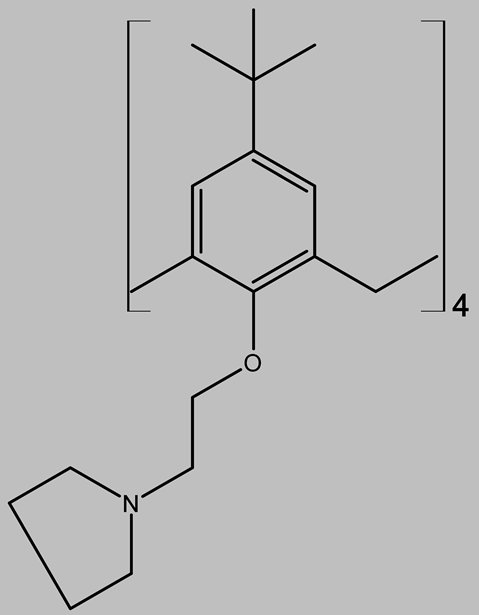
**19f**	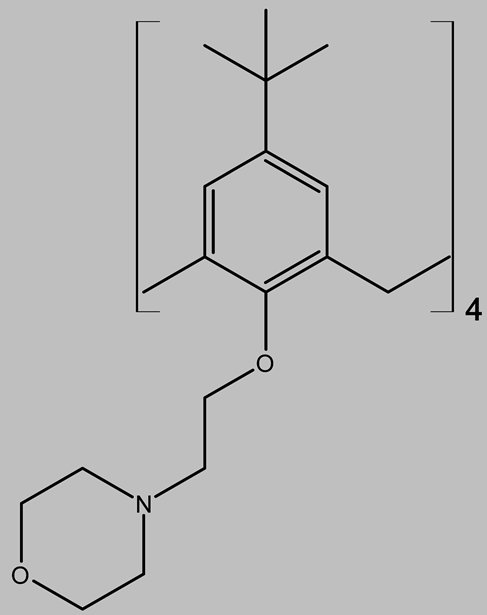	**19g**	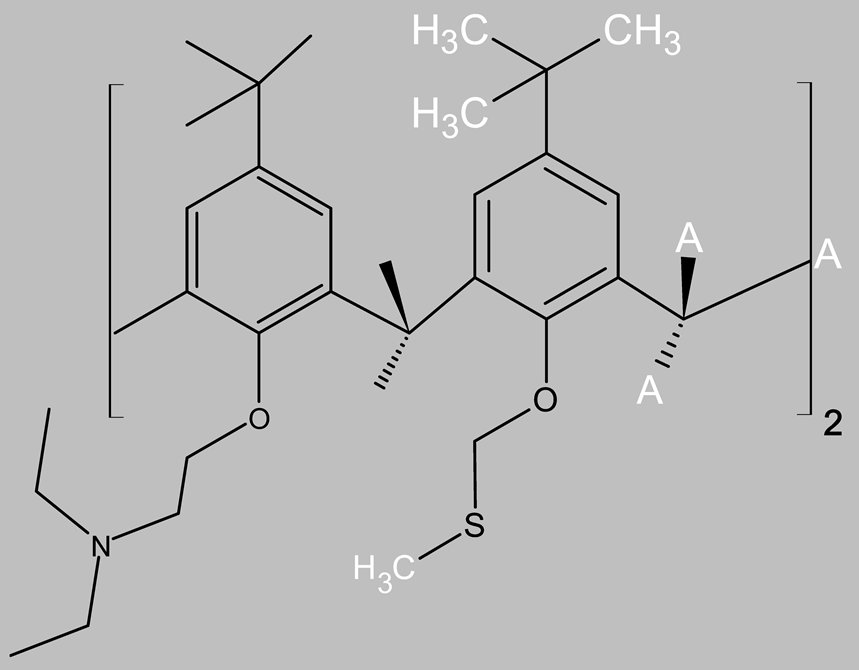
**20**	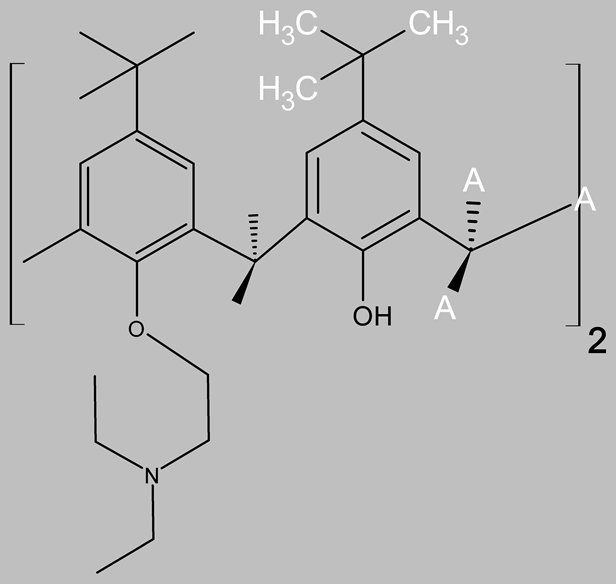	**21**	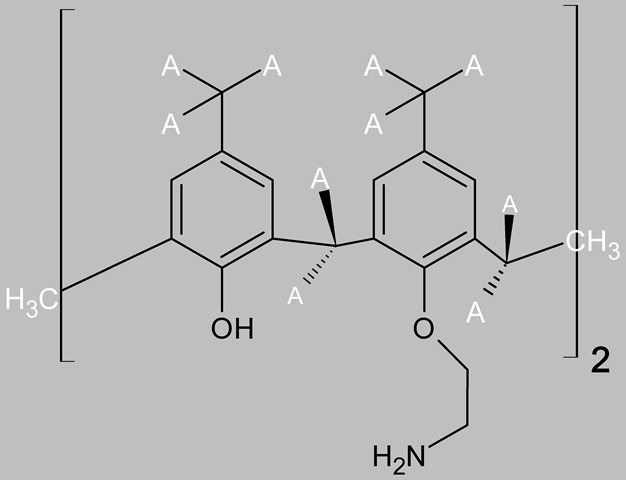
**22a**	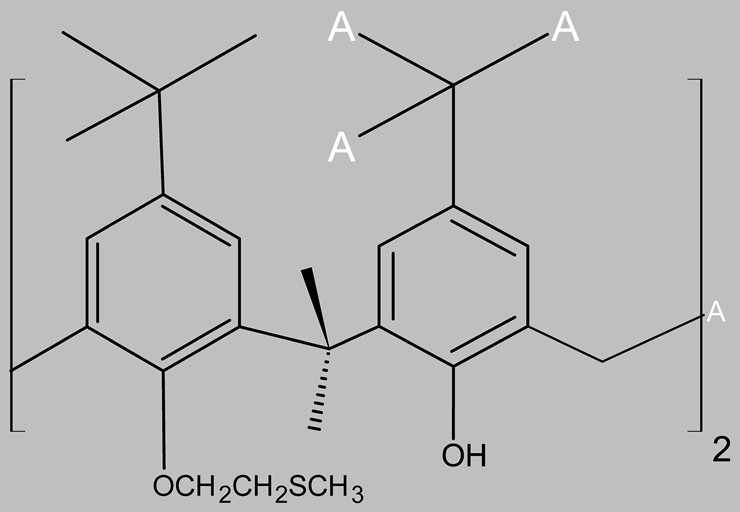	**22b**	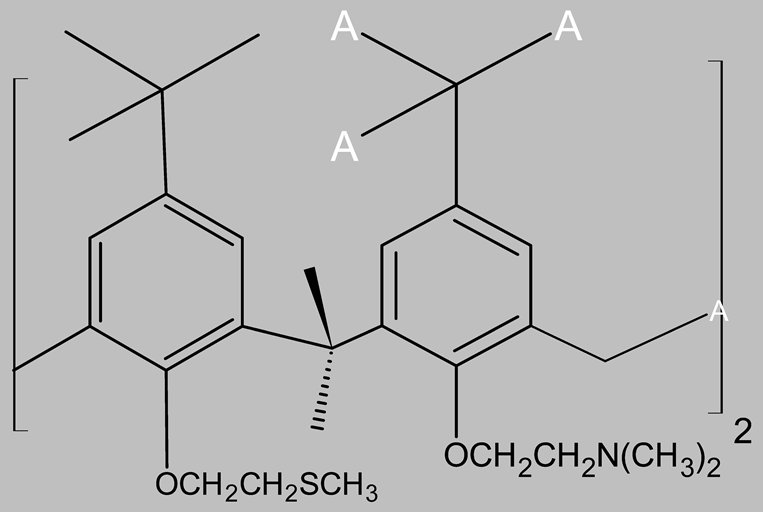
**22c**	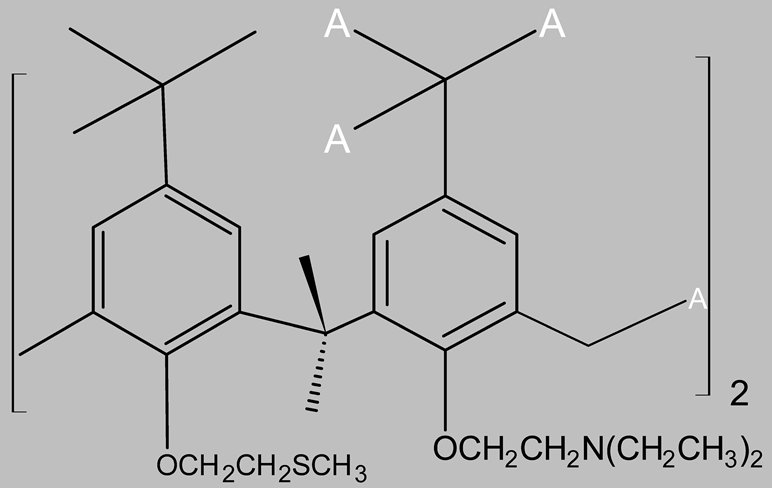	**22d**	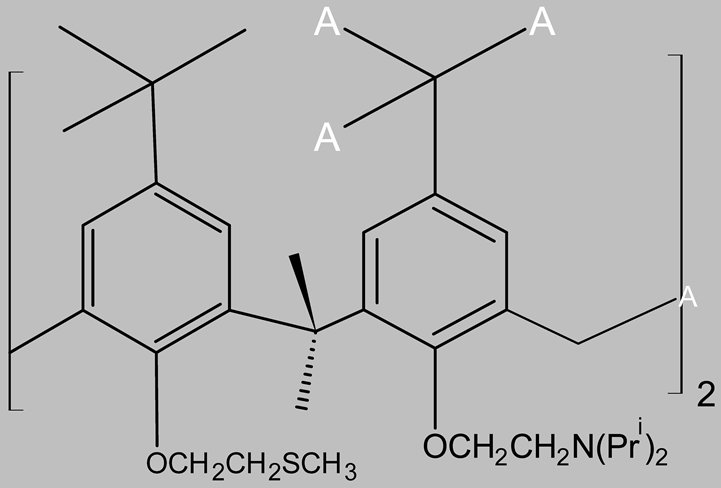
**22e**	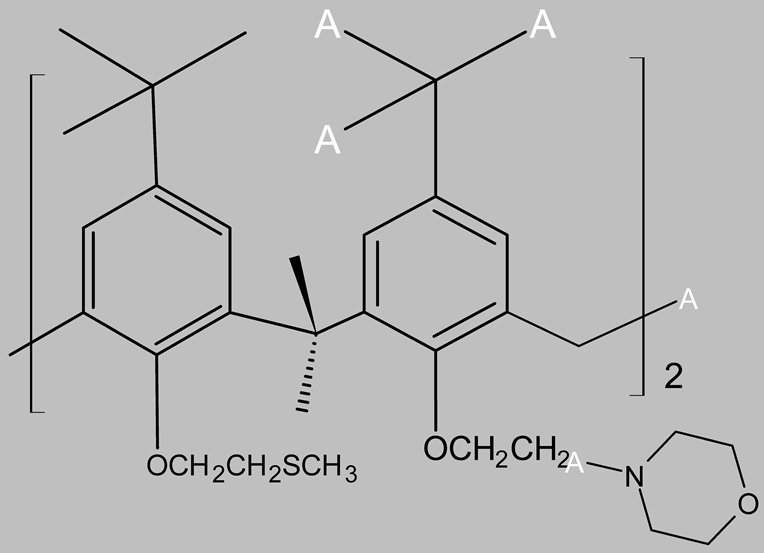	**22f**	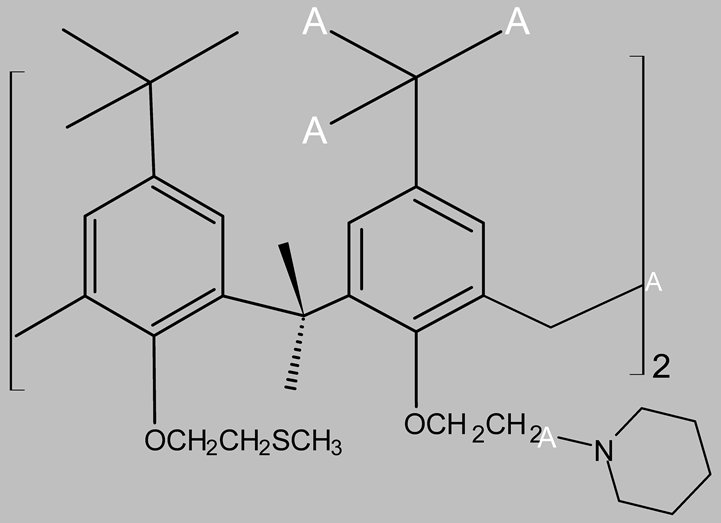
**22g**	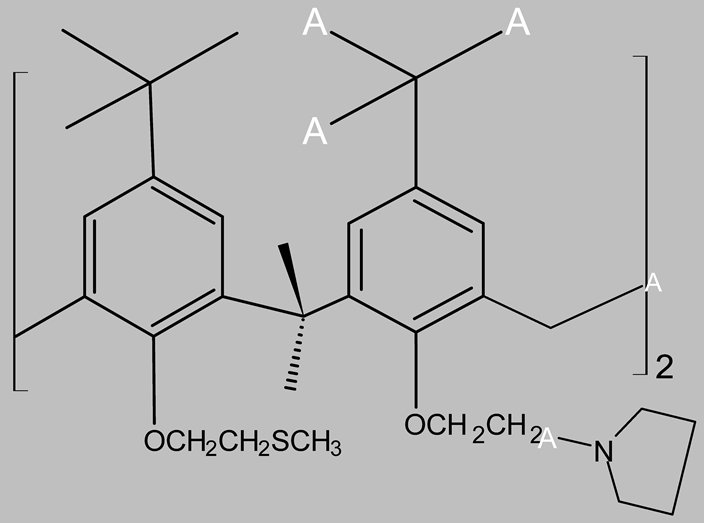	**22h**	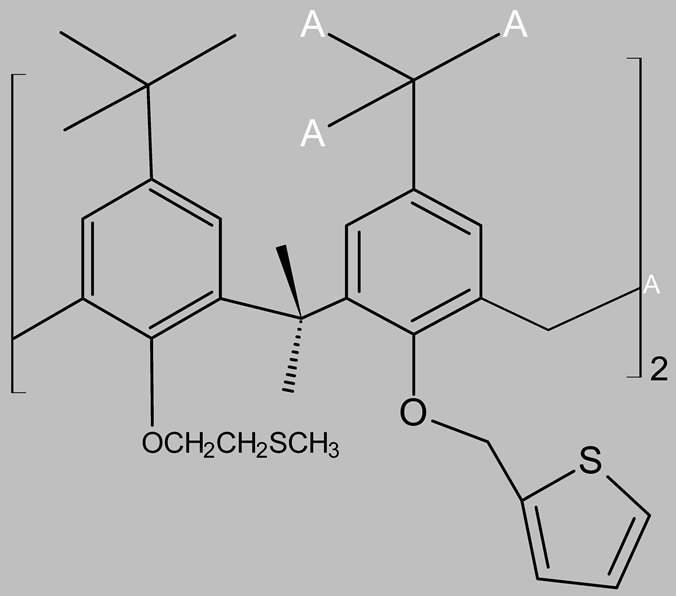
**22i**	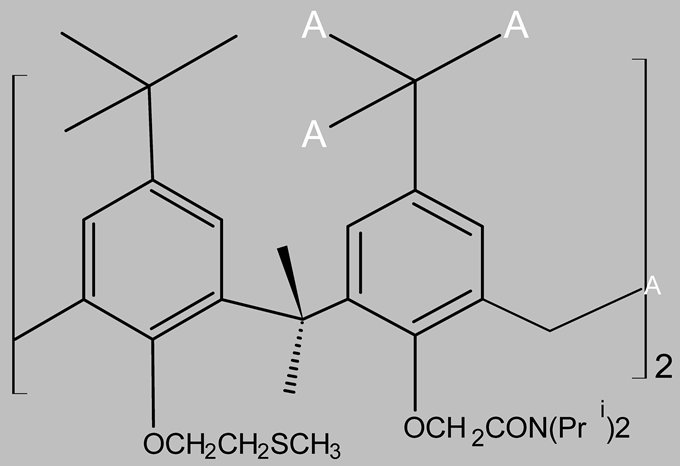	**22j**	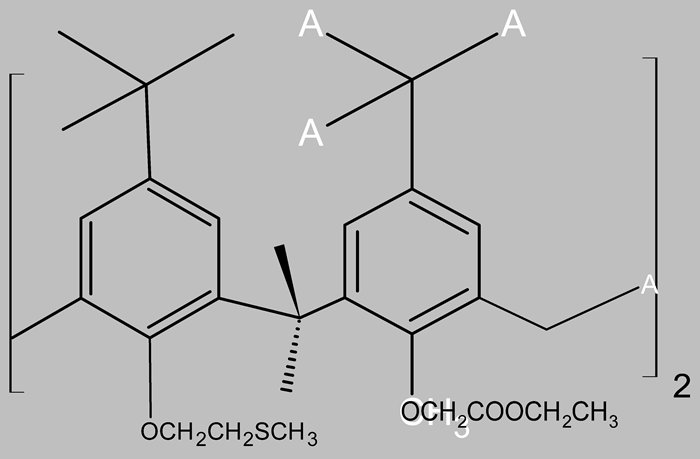
**23**	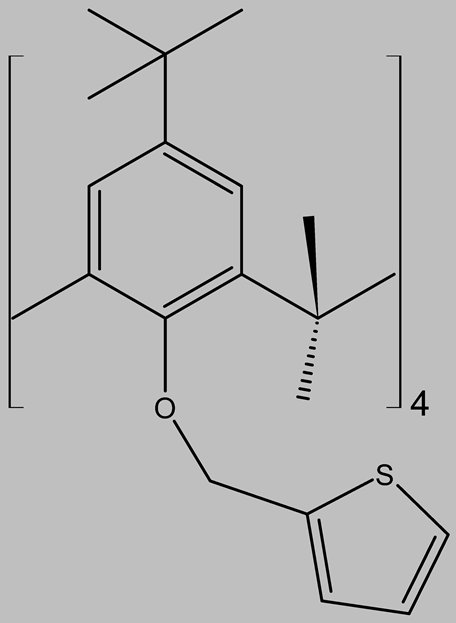	**24**	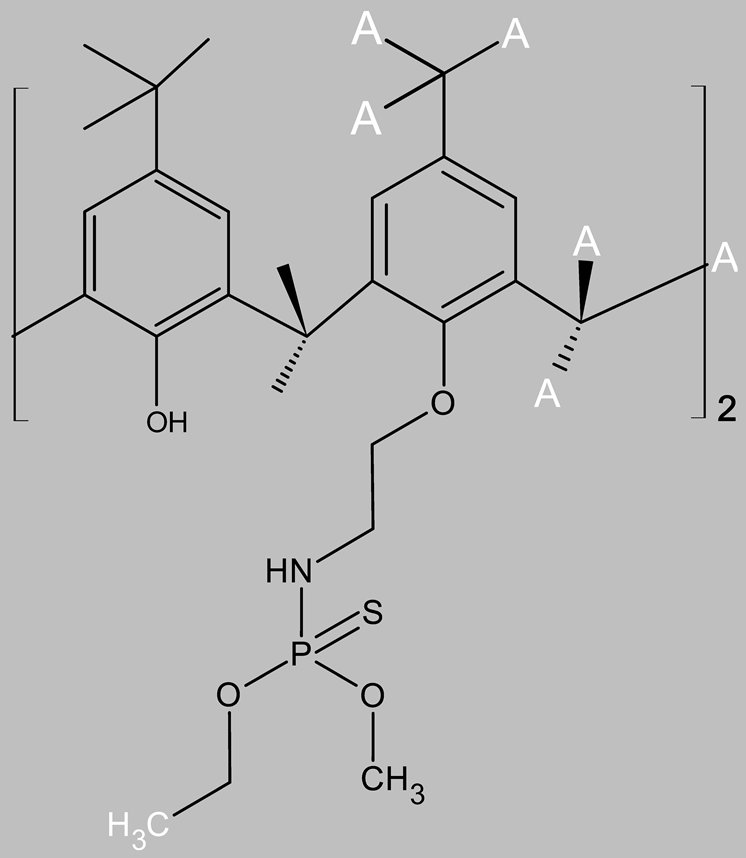
**25**	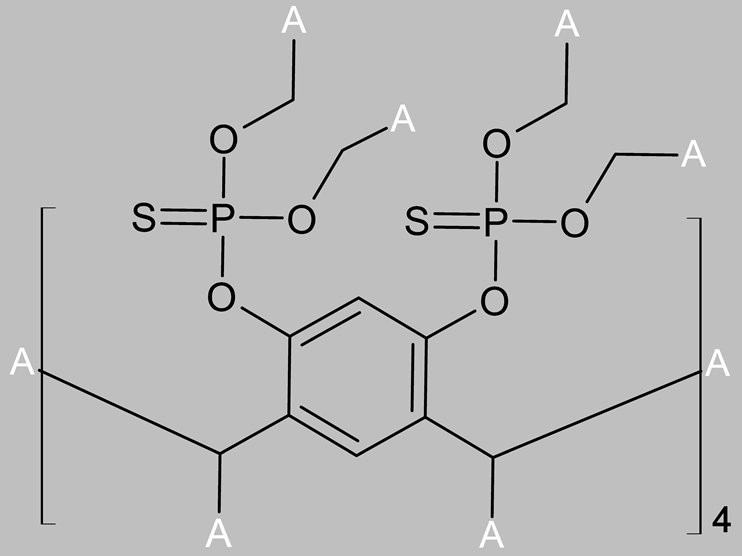	**26**	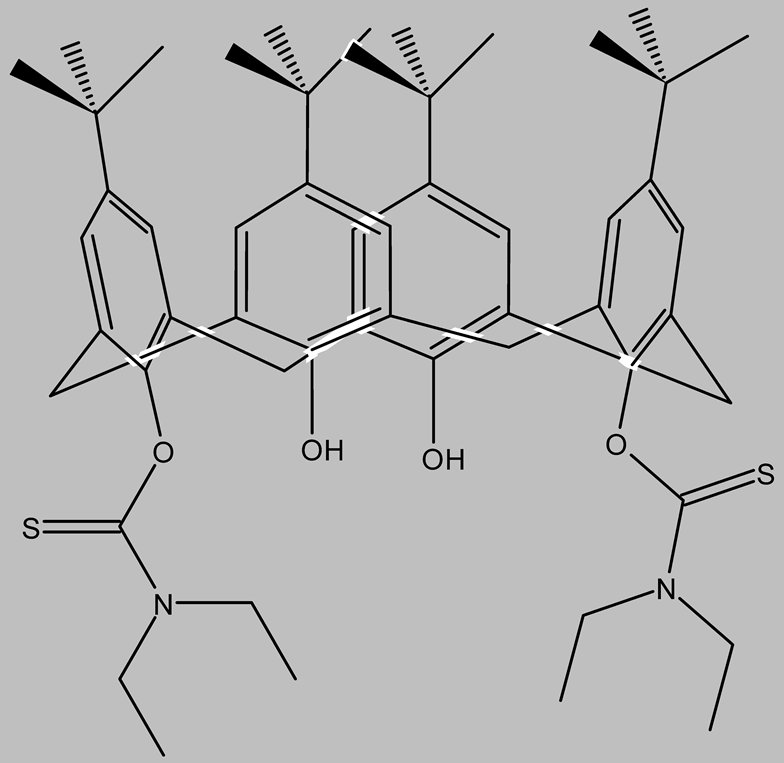
**27**	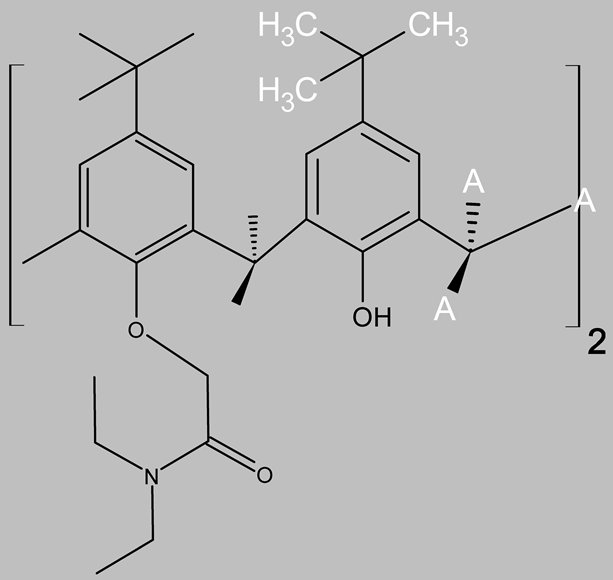	**28**	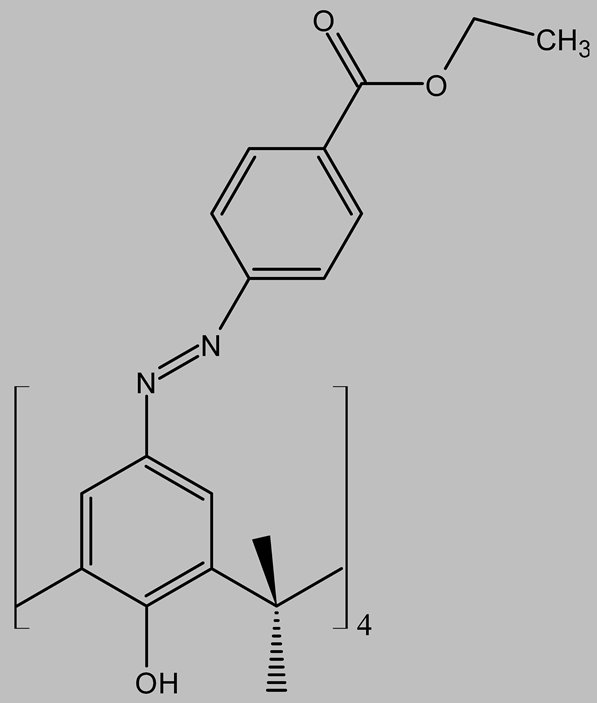
**29**	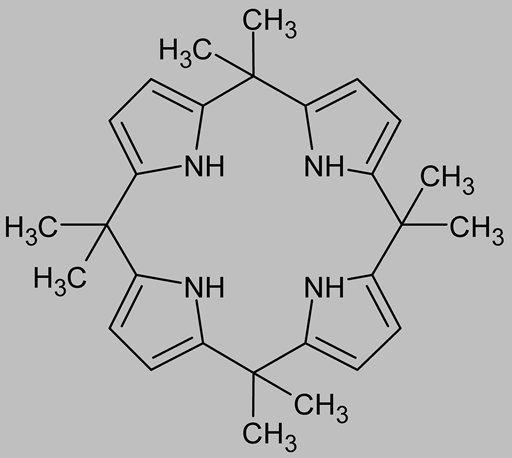	**30**	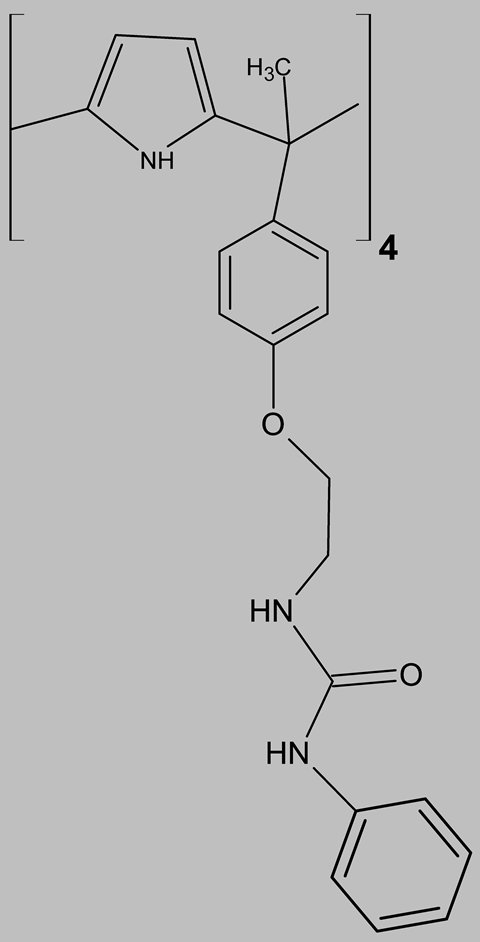
**31a**	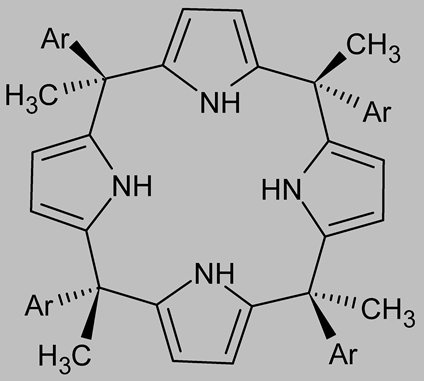 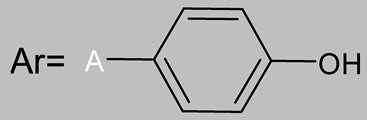 αβαβ	**31b**	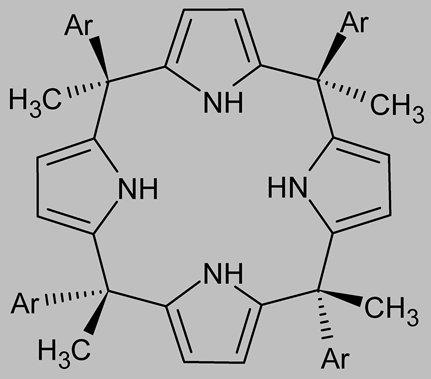 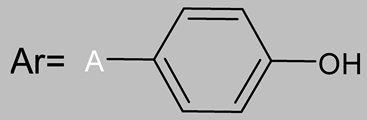 ααββ
**32**	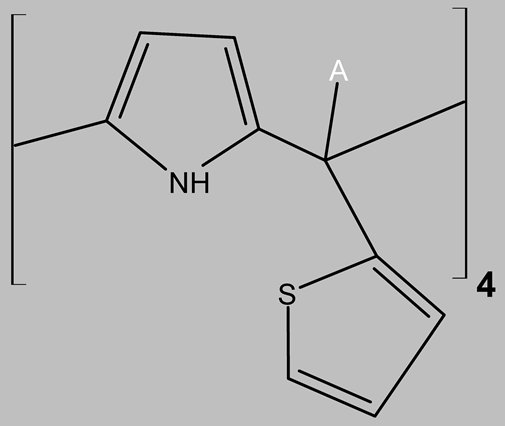	**33**	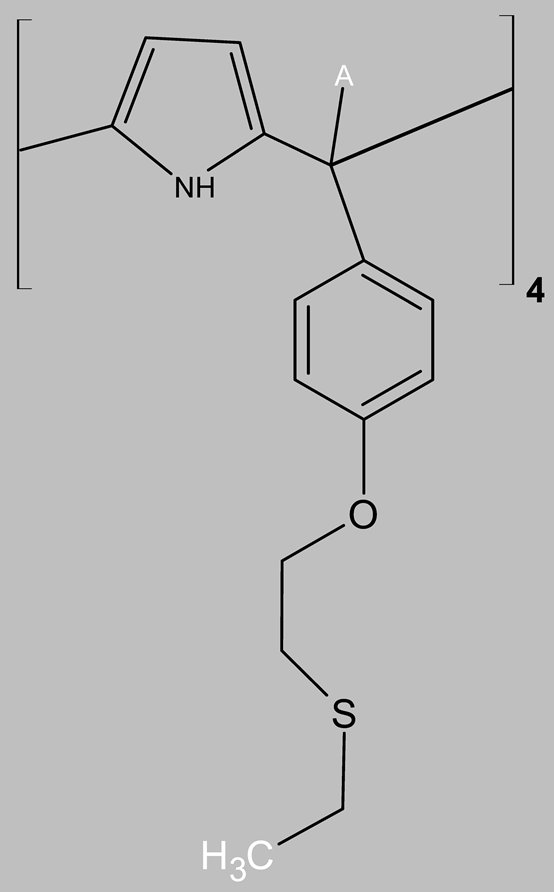
**34**	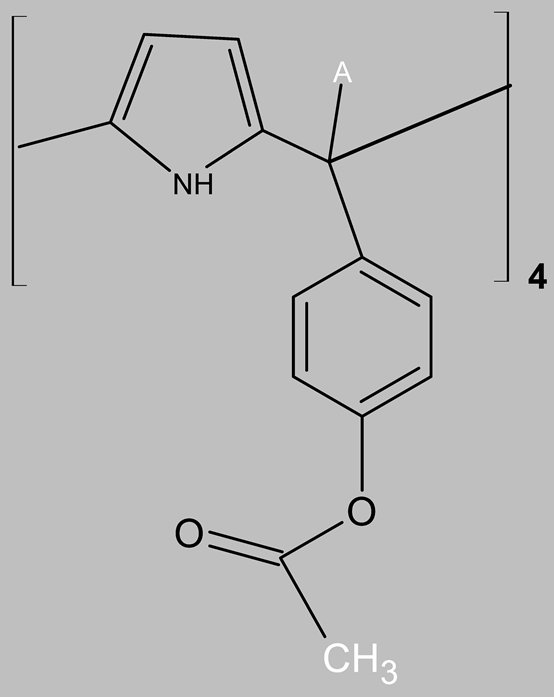	**35**	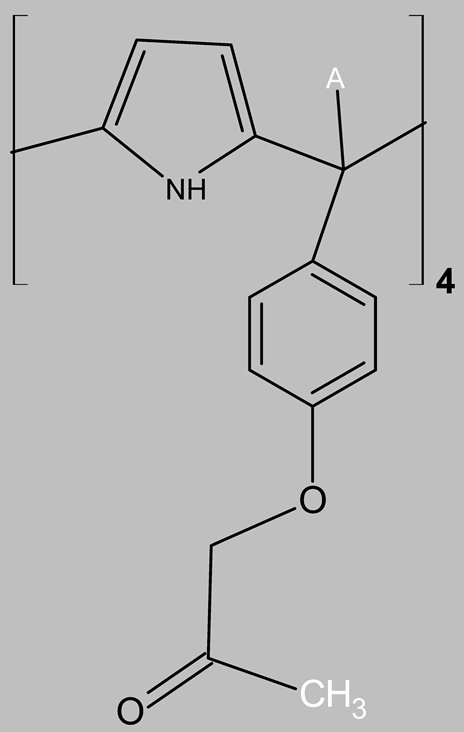
**36**	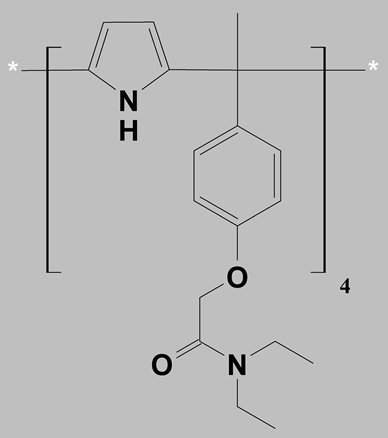	**37**	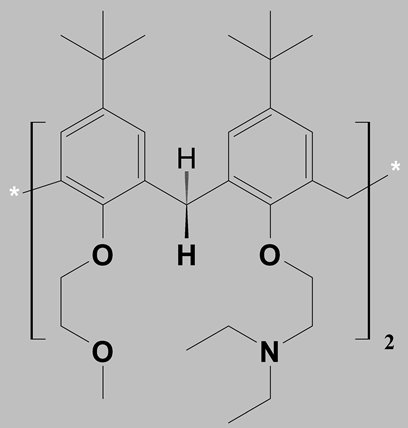
**38**	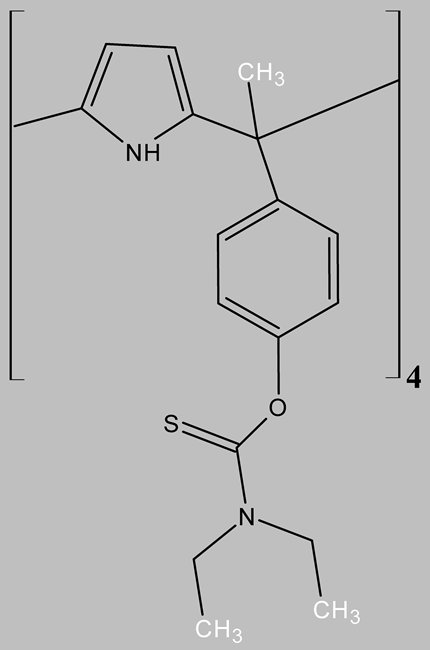	**39**	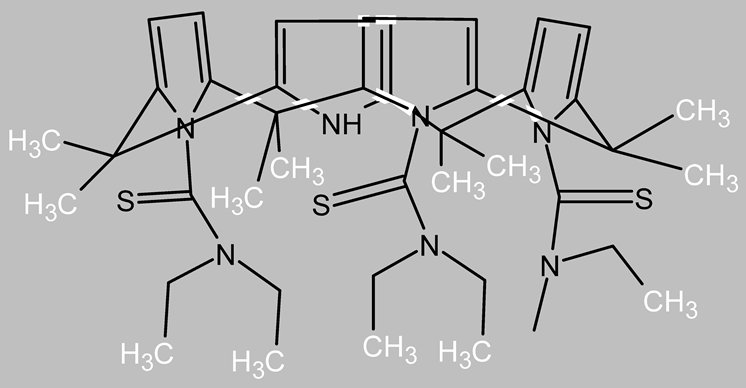
**40a**	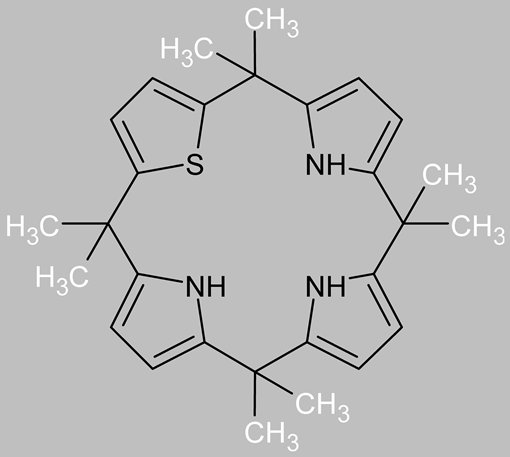	**40b**	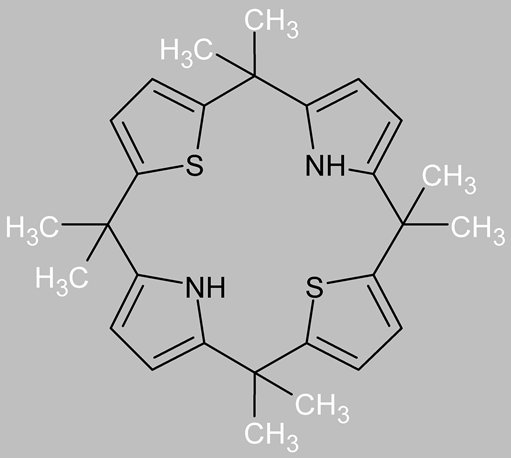
**40c**	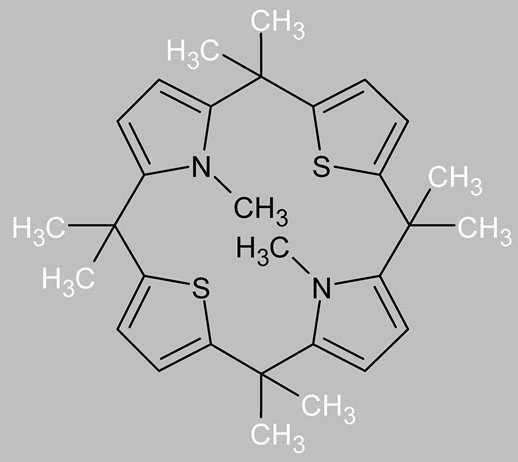	**41**	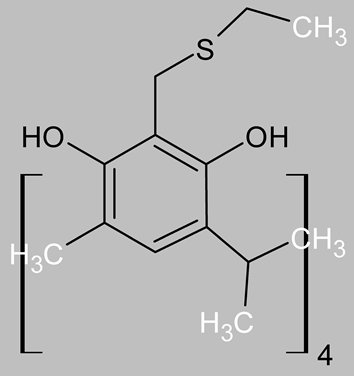
**42**	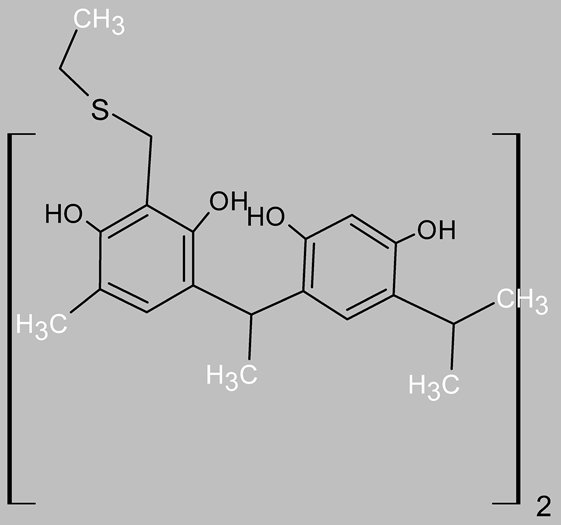	**43**	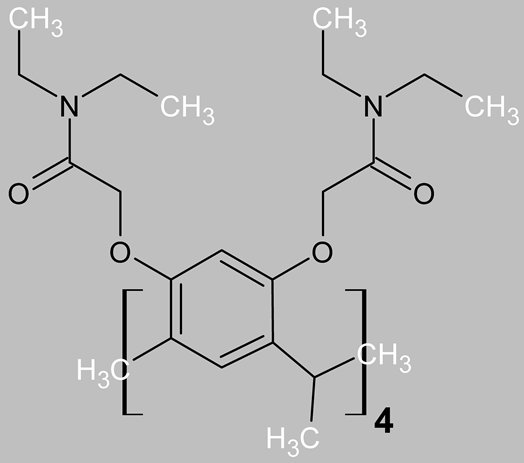

## Data Availability

New data were created or analyzed in this study. Data sharing is not applicable to this article.
